# Increased intrinsic excitability of muscle vasoconstrictor preganglionic neurons may contribute to the elevated sympathetic activity in hypertensive rats

**DOI:** 10.1152/jn.00350.2014

**Published:** 2014-08-13

**Authors:** Linford J. B. Briant, Alexey O. Stalbovskiy, Matthew F. Nolan, Alan R. Champneys, Anthony E. Pickering

**Affiliations:** ^1^School of Physiology and Pharmacology, University of Bristol, Bristol, United Kingdom;; ^2^Department of Anaesthesia, University Hospitals Bristol, Bristol, United Kingdom;; ^3^Department of Engineering Mathematics, University of Bristol, Bristol, United Kingdom; and; ^4^Centre for Integrative Physiology, University of Edinburgh, Edinburgh, United Kingdom

**Keywords:** sympathetic preganglionic, vasomotor tone, hypertension, transient rectification

## Abstract

Hypertension is associated with pathologically increased sympathetic drive to the vasculature. This has been attributed to increased excitatory drive to sympathetic preganglionic neurons (SPN) from brainstem cardiovascular control centers. However, there is also evidence supporting increased intrinsic excitability of SPN. To test this hypothesis, we made whole cell recordings of muscle vasoconstrictor-like (MVC_like_) SPN in the working-heart brainstem preparation of spontaneously hypertensive (SH) and normotensive Wistar-Kyoto (WKY) rats. The MVC_like_ SPN have a higher spontaneous firing frequency in the SH rat (3.85 ± 0.4 vs. 2.44 ± 0.4 Hz in WKY; *P* = 0.011) with greater respiratory modulation of their activity. The action potentials of SH SPN had smaller, shorter afterhyperpolarizations (AHPs) and showed diminished transient rectification indicating suppression of an A-type potassium conductance (*I*_A_). We developed mathematical models of the SPN to establish if changes in their intrinsic properties in SH rats could account for their altered firing. Reduction of the maximal conductance density of *I*_A_ by 15–30% changed the excitability and output of the model from the WKY to a SH profile, with increased firing frequency, amplified respiratory modulation, and smaller AHPs. This change in output is predominantly a consequence of altered synaptic integration. Consistent with these in silico predictions, we found that intrathecal 4-aminopyridine (4-AP) increased sympathetic nerve activity, elevated perfusion pressure, and augmented Traube-Hering waves. Our findings indicate that *I*_A_ acts as a powerful filter on incoming synaptic drive to SPN and that its diminution in the SH rat is potentially sufficient to account for the increased sympathetic output underlying hypertension.

sympathetic activity is elevated in hypertensive patients in prehypertensive conditions and in animal models of hypertension (reviewed in Esler 2011; Fisher and Paton 2012; Grassi 1998). This has recently led to trials of novel therapeutic interventions aimed at reducing the sympathetic overactivity, for example, renal nerve denervation (Schlaich et al. 2009) and carotid sinus stimulation (Jordan et al. 2012). Notwithstanding these advances, hypertension remains a common clinical problem, and despite a range of drug treatments a substantial population of hypertensive patients (∼14%) remain refractory to therapy and at risk of cardiovascular morbidity (Carey 2013). Therefore, there is an imperative to better understand the factors leading to the increased sympathetic outflow.

The sympathetic outflow is specialized according to the target organs, and the muscle vasoconstrictor class (MVC) of sympathetic neuron is believed to be particularly important in the control of blood pressure (Janig 2006). These sympathetic vasoconstrictor pathways produce a tonic release of norepineprine that maintains vascular tone. This tonic sympathetic activity originates in supraspinal structures including the rostro-ventrolateral medulla (RVLM) and the hypothalamic paraventricular nucleus (reviewed in Guyenet 2006). Mechanisms suggested to account for the elevation of sympathetic nerve activity (SNA) seen in hypertension have focused on these brainstem and higher centers (Moraes et al. 2014; Sved et al. 2003) or on cardiorespiratory afferent inputs (DiBona and Esler 2010; McBryde et al. 2013). Intriguingly, there have been reports of increased sympathetic excitability at a spinal level in spontaneously hypertensive rats that is maintained after removal of inputs to the spinal cord (Schramm and Barton 1979; Schramm and Chornoboy 1982; Schramm et al. 1979). As yet the cellular mechanisms for this spinally mediated increase in sympathetic discharge have received relatively little attention.

The intrinsic membrane properties of sympathetic preganglionic neurons (SPN) may be important determinants of the sympathetic activity received by blood vessels as they have powerful rectifying conductances, including a prominent transient rectification, *I*_A_ (Dembowsky et al. 1986; Miyazaki et al. 1996; Pickering et al. 1991; Whyment et al. 2011; Yoshimura et al. 1987), that contribute to their relatively low (2–3 Hz) firing frequency in response to the high frequency (>20 Hz) of ongoing synaptic inputs (Stalbovskiy et al. 2014). We therefore set out to investigate the possibility that altered excitability of SPN accounts for increased sympathetic activity in hypertension. To test this hypothesis we obtained whole cell recordings of MVC_like_ SPN in the working heart brainstem preparation (Paton 1996), wherein the SPN can be functionally characterized by their responses to cardiorespiratory reflex activation (Stalbovskiy et al. 2014). This allowed us to characterize both the intrinsic properties and network drives of SPN in spontaneously hypertensive (SH) rats and also normotensive Wistar-Kyoto (WKY) rats. We undertook these studies in neonatal animals (P7–16), before they have developed hypertension, allowing us to detect changes in excitability that could be causal rather than simply associative.

We find that the firing frequency of MVC_like_ SPN is increased in the SH rat with an exaggerated respiratory-sympathetic modulation, findings that echo the whole sympathetic nerve recordings of Simms et al. (2010; 2009). This is associated with a diminution in their transient rectification but no apparent change in the incoming synaptic input to SPN. We therefore built a conductance-based model of a MVC_like_ SPN in the NEURON environment (Hines et al. 2004) with particular focus on achieving biophysically accurate kinetics of *I*_A_ (Bordey et al. 1995; Whyment et al. 2011). We show that varying the conductance density of *I*_A_ replicates the increased sympathetic output and altered excitability seen in our recordings without a requirement for a change in the afferent drive. Furthermore, we show that intrathecal administration of 4-aminopyridine (4-AP) to block the A-current at a spinal level in situ (Pickering and Paton 2006; Sadananda et al. 2011) produces a dramatic increase in the level of sympathetic activity consistent with it playing a substantial role in gating the sympathetic outflow.

## EXPERIMENTAL METHODS

All experiments conformed to the UK Home Office guidelines regarding the ethical use of animals and were approved by the University of Bristol Ethical Review Committee. Male WKY rats (*n* = 34, P7–16) and SH rats (Okamoto and Aoki 1963; *n* = 32, P8–16) were used in the cell recording studies, and WKY rats (*n* = 6, P21–24) were used for the sympathetic nerve recordings.

### Working Heart Brainstem Preparation

The working heart brainstem preparation (WHBP) was used for all patch-clamp recordings of SPN in the lateral horn of the spinal cord (Stalbovskiy et al. 2014). In brief, rats were deeply anesthetized with halothane until loss of withdrawal to paw pinch. The rat was bisected subdiaphragmatically, exsanguinated, cooled in Ringer's solution at 5°C, and suction decerebrated precollicularly following which the halothane anesthesia was discontinued. The preparation was kept cold while the phrenic nerve and descending aorta were dissected free and a bilateral pneumonectomy was performed.

The preparation was positioned prone while still cold and access to the spinal cord was obtained via a laminectomy up to the level of C7. The dura was incised and the dorsal pia mater was removed locally at the level of T3. A single cut was made in the spinal cord using a custom-built piezoslicer (Smith et al. 2007). This employed a piezoelectric bending actuator (Piezo Systems, Woburn, MA) with a microblade (FST 10035-05) to produce the “slice in situ” preparation with a 45° bevel on the cut end of the cord at the level of T3 for recordings.

The preparation was transferred to a recording chamber in ear bars and positioned prone to allow access to the cut surface of the spinal cord slice. A double lumen cannula (Ø 1.25 mm, DLR-4; Braintree Scientific) was inserted into the descending aorta for retrograde perfusion with carbogen-gassed, modified Ringer's solution (see below for composition) containing Ficoll-70 (1.25%; Sigma) at 30°C. The perfusion pressure was monitored via the second lumen of the cannula. The heart resumed beating almost immediately as the perfusate flow was commenced (11–13 ml/min), and rhythmic respiratory muscle contractions commenced after 1–3 min, signaling the return of brainstem function. At this point muscle relaxant was added to the perfusion solution (200 mcg vecuronium; Norcuron; Organon, Cambridge, UK) to allow stable recordings.

Phrenic nerve activity was recorded using a glass suction electrode to give a physiological index of preparation viability. The signal from the phrenic nerve was AC amplified and band-pass filtered (80-3 kHz). The perfusion pressure was adjusted to obtain an optimal eupnoeic pattern of PNA by addition of vasopressin (2–400 pM; Sigma) to the reservoir and/or increase of the pump flow rate. Chlorided silver electrodes were inserted bilaterally into the rib cage to record ECG allowing instantaneous heart rate to be derived.

### Decerebrate Arterially Perfused Rat Preparation

The decerebrate arterially perfused rat (DAPR) preparation was used to examine the effect of intrathecal 4-AP upon the sympathetic outflow and was set up using previously described methods (Pickering and Paton 2006; Sadananda et al. 2011). In brief, WKY rats (40–90 g, P21–24) were heparinized (100 IU ip) before being deeply anesthetized with halothane, until loss of withdrawal to paw pinch. Following a midline laparotomy, the stomach, spleen, and free intestine were vascularly isolated with ligatures and removed. The animal was immediately cooled by immersion in Ringer's (5°C, composition below) and decerebrated, by aspiration, at the precollicular level to render it insentient (at this point the halothane was withdrawn).

After skin removal and a midline sternotomy, the thoracic cavity was opened with insertion of a spreading retractor. The left phrenic nerve was identified, and the lungs and diaphragm were removed. Both atria were incised to avoid venous congestion during subsequent arterial perfusion. An incision was made at the apex of the heart for insertion of the perfusion cannula into the ascending aorta. A single segment laminectomy allowed an intrathecal 32-gauge intrathecal catheter (CR3212; ReCathCo; Allison Park, PA) to be threaded through a 25-gauge hypodermic needle under direct vision to sit at a low thoracic level.

The preparation was transferred to the recording chamber, and a double lumen cannula was inserted into the ascending aorta. The preparation was arterially perfused (flow rate: ∼30 ml/min) and optimized, and the phrenic nerve was recorded as for the WHBP (above). Recordings from the thoracic sympathetic chain were obtained using a bipolar suction electrode at the level of T12 and were AC amplified and band-pass filtered (100 Hz to 3 kHz).

### Whole Cell Recordings from Sympathetic Preganglionic Neurons

The outline of the lateral horn was clearly visible under a binocular microscope (Leica MZ-6) on the cut face of the cord, allowing the recording patch electrode to be directed into the SPN cell column. Blind, whole cell recordings were made from neurons at depths of 50–500 μm below the cut surface. Electrodes were pulled from borosilicate capillaries (GC150-TF10; Harvard Apparatus) to have a resistance of 5–10 MΩ. Stable gigaohm seals and subsequent whole cell recordings were obtained from neurons for periods of over 1 h with access resistances of 20–50 MΩ.

Current-clamp and voltage-clamp recordings were made using a discontinuous clamp amplifier (SEC-05LX-BF; npi electronic, Tamm, Germany) with switching frequencies between 10 and 15 kHz and a 25% duty cycle after optimization of capacitance compensation. The gain was maximally increased to just below the point of clamp instability (typically ∼1,000×) as assessed from continuous monitoring of the electrode potential output. Cell recordings were low-pass filtered at 2 kHz, and the signal was passed through a Humbug (Digitimer) to remove mains interference. Data were sampled at 5 kHz using a power1401 A–D converter (CED).

Lateral horn neurons were definitively identified as being SPN by antidromic activation following stimulation (0.3–1 ms, 5–20 V, 0.2–20 Hz) of the ventral root exit zone of the spinal cord with a concentric bipolar electrode (SNE100; Rhodes Medical Instruments). Cancellations were sought by depolarizing the cell to fire spontaneous action potentials while applying ventral root stimuli. A total of 43/64 (67.2%) of the SPN tested were definitively identified antidromically. The remaining cells were identified as SPN on the basis of characteristic electrophysiology, post hoc anatomical reconstruction, and their responses to functional cardiorespiratory reflex activation (Stalbovskiy et al. 2014).

### Cardiorespiratory Reflexes

In each neonatal WHBP preparation the afferent stimulus was titrated at the start of the experiment to reproducibly evoke the expected physiological responses. Peripheral chemoreceptors were stimulated using intra-arterial injection of sodium cyanide (50–100 μl of 0.03%) as a bolus into the perfusion line. The chemoreflex responses were dose dependent, and the doses used produced submaximal bradycardia (1–2 Hz) and hyperpnea. The diving response was evoked by application of cold Ringer's (∼10°C, 50–200 μl) to the snout that triggered a characteristic apnea (lasting for >2× basal respiratory cycle period) and transient bradycardia.

### SPN Recording Protocol

The following seal rupture the initial recordings were made in current-clamp mode and the cell was allowed to stabilize before

*1*) baseline current-clamp recording of firing activity (∼1 min);

*2*) diving response and peripheral chemoreflex activation;

*3*) voltage responses to injection of current pulses;

*4*) antidromic stimulation; and

*5*) voltage clamp to resolve synaptic events.

#### Experimental data recording and analysis.

Perfusion pressure, electrocardiogram, and phrenic nerve activity were recorded using custom built AC amplifiers and transducers (designed and built by Jeff Croker, University of Bristol) and collected via an A–D interface (power1401; CED, Cambridge, UK) to a computer running Spike2 software (CED). Custom scripts were used for data acquisition and analysis in Spike2.

All membrane potentials were corrected for a junction potential of 13 mV. SPN with resting membrane potentials greater than −40 mV and whose action potentials overshot zero were included for the analysis of membrane properties. Spike parameters were measured from spontaneous action potentials, and the threshold for spike discharge was taken arbitrarily as the point at which the rate of rise of membrane potential exceeded 7.5 V·s^−1^. The spike parameters were measured with reference to this threshold point. Spike amplitude was measured above threshold and duration was calculated at 1/3 of spike amplitude. The duration of the afterhyperpolarization (AHP) was calculated from where spike repolarization crossed threshold to the point of return to resting potential. The AHP amplitude was measured from spike threshold to the trough. The input resistance and time constant were estimated from the voltage deflection (amplitude: 5–10 mV) in response to small hyperpolarizing current pulses (5–20 pA applied for 1 s).

#### Statistical analysis.

Data are expressed as means ± SE or median [interquartile range]; *n* refers to the number of cells. Two tailed *t*-tests or ANOVA were used to establish statistical significance (Prism 5; GraphPad Software, San Diego, CA) defined as *P* < 0.05.

#### Drugs and solutions.

The composition of the modified Ringer's solution used as perfusate was as follows (in mM): 125 NaCl, 24 NaHCO_3_, 3 KCl, 2.5 CaCl_2_, 1.25 MgSO_4_, 1.25 KH_2_PO_4_, and 10 dextrose pH 7.35–7.4 after carbogenation. The patch solution contained the following (in mM): 130 K-gluconate, 10 KCl, 10 NaCl, 2 MgCl_2_, 10 HEPES, 2 NaATP, and 0.2 NaGTP (pH 7.4 and osmolarity of 300 mosM). All chemicals were from Sigma.

## COMPUTATIONAL OVERVIEW

A quantitative model of a MVC_like_ SPN was constructed within the simulation environment NEURON v7.3 (Carnevale 2006); code for model now deposited on ModelDB (senselab.med.yale.edu/modeldb; Accession No. 151482).

### Model Cell Morphology

The model SPN (see Fig. 4) was based on experimental data (Forehand 1990; Sah and McLachlan 1995) and had an ovoid soma with dimensions 25 × 15 μm with three lateral dendrites (200-μm long × 2-μm diameter, 10 segments) and a single medial primary dendrite (25 × 5 μm, 5 segments) from which two secondary medial dendrites emerge (600 × 2 μm, 20 segments). A single unbranched axon arises from the soma (length of 500 μm and diameter of 0.5 μm, 20 segments). The axial resistance was 120 Ω·cm, and the membrane capacitance was 1 μF/cm^2^.

### Membrane Properties

The passive electrophysiological properties of SPN in the rat have been reported from numerous in vitro studies, with resting membrane potentials of approximately −55 mV (Logan et al. 1996; Miyazaki et al. 1996; Pickering et al. 1991; Sah and McLachlan 1995; Whyment et al. 2011; Wilson et al. 2002; Yoshimura et al. 1986a,b). The reversal potential and maximal conductance density of the leak conductance were set to *E*_pas_ = −40 mV and *g*_pas_ = 0.018 mS·cm^−2^ in the soma to adjust both the resting membrane potential and input resistance to be within the physiological range. The input resistance (*R*_in_) was measured as 320.9 MΩ in keeping with experimental data (Sah and McLachlan 1995; Stalbovskiy et al. 2014; Wilson et al. 2002).

The model included passive, voltage-dependent and calcium-dependent conductances (Fig. 4) selected based on experimental evidence for their involvement in determining membrane excitability close to the resting potential (see Table A1 in appendix). The parameters for the voltage-gated channels (see appendix for full descriptions) were based on previously published experimental and modeling studies (Migliore et al. 1995, 1999). This study focused on the influence of the A-current on SPN excitability across the rat strains and as such the parameters for this conductance were fitted to the existing experimental data (Dembowsky et al. 1986; Miyazaki et al. 1996; Sah and McLachlan 1995; Yoshimura et al. 1987) and particularly the in depth characterization by Whyment et al. (2011) and Bordey et al. (1995) (see appendix). All active conductances were present in the soma. The leak current (*I*_pas_) was present throughout the cell. The axon had the Hodgkin-Huxley conductances required for spike generation (*I*_Na3_, *I*_DR_). The dendrites were passive.

### Model Simulation

Simulations of our single-cell model were performed on a two dual-core Opteron processors 8GB RAM node, using the computational facilities of the Advanced Computing Research Centre, University of Bristol (http://www.bris.ac.uk/acrc/). Simulation data were imported into MATLAB 6.1 (2000; The MathWorks, Natick, MA) for analysis and graphing on a personal desktop (Toshiba Tecra). Statistical tests were conducted in Prism v2.0 (GraphPad). As well as looking at the output from the model in simulated current- and voltage-clamp modes, it was also driven in a more physiological mode with experimentally recorded synaptic currents to generate spike activity. These data were obtained from 50-s voltage-clamp recordings from MVC_like_ SPN.

## RESULTS

To compare the electrophysiological properties of SPN from SH and WKY rats, we obtained whole cell recordings from neurons in the working heart brainstem preparation (Paton 1996). A total of 90 SPN (*n* = 50 WKY, *n* = 40 SH) were recorded from 66 WHBP (32 from SH and 34 from WKY rats). SPN were classified on the basis of their responses to cardiorespiratory reflex activation (Stalbovskiy et al. 2014). We identified the MVC_like_ class of SPN by their excitatory responses to peripheral chemoreflex activation and to diving response initiation (Stalbovskiy et al. 2014). The dataset reported included 22 MVC_like_ SPN from WKY and 23 MVC_like_ SPN from age-matched SH rats (postnatal days 12.7 ± 0.5 vs. 11.4 ± 0.5, respectively; *P* = 0.7).

### Increased Spontaneous Activity of MVC_like_ SPN in SH Rats

**Fig. 1.**
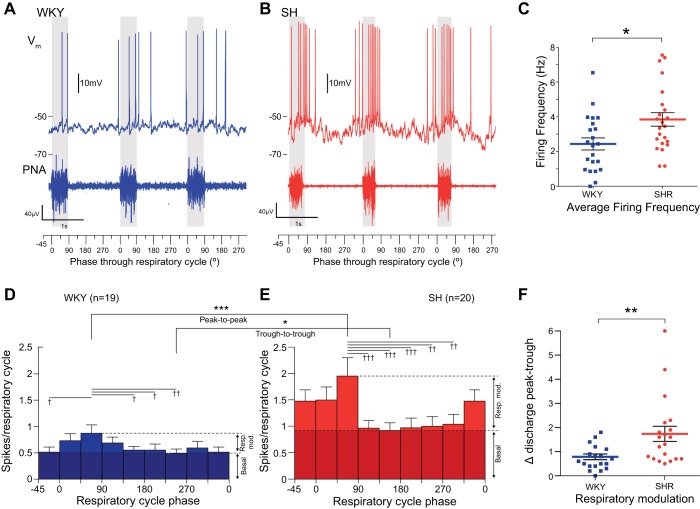
Increased activity of muscle vasoconstrictor (MVC_like_) sympathetic preganglionic neurons (SPN) in spontaneously hypertensive (SH) rats. MVC_like_ SPN of Wistar-Kyoto (WKY) (*A*) and SH rats (*B*) both exhibited respiratory modulation of discharge entrained to phrenic nerve activity (PNA), but the SH SPN has an increased firing frequency with larger respiratory modulated bursts occurring in the I and PI phase. *C*: MVC_like_ SPN of the SH rat had a higher mean firing frequency [SH 3.85 ± 0.39 Hz (*n* = 23) vs. WKY 2.44 ± 0.35 Hz, WKY (*n* = 22); *P = 0.01, *t*-test]. *D* and *E*: phase histograms of MVC_like_ SPN discharge across strains showed a pattern of respiratory modulation of activity (firing activity over the respiratory cycle apportioned into eight 45° bins; WKY *n* = 19, SH *n* = 20 SPN, activity averaged from 10 respiratory cycles for each cell). The grouped WKY MVC_like_ SPN activity had clear respiratory modulation (one-way ANOVA, *P* = 0.002; *n* = 19) with a peak of discharge in the 45° bin (I phase; ††*P* < 0.01) compared with the trough during 225° (ME phase) and also the 135°, 180°, and −45/315° bins (†*P* < 0.05). *E*: similarly, the SH MVC_like_ SPN also showed respiratory modulation (one-way ANOVA, *P* < 0.001; *n* = 20) with a peak at 45° compared with the trough at 135° (†††*P* < 0.001). The trough now begins 90° earlier (compared to WKY), and the ramp up in activity to the peak starts earlier in the cycle. Comparison across strains showed both strain and phase were significant sources of variation [phase (*P* < 0.0001) and strain (*P* < 0.01) with an interaction *P* < 0.01, two-way mixed measures ANOVA] with a significant increase in the peak seen particularly in the 45° bin in the SH rat (***P < 0.001, Bonferroni post hoc test). Post hoc testing also showed higher basal firing level (shaded) in the SH rats (trough-to-trough, *P < 0.05, *t*-test). *F*: degree of respiratory modulation of MVC_like_ activity as peak-to-trough difference in spike count across the bins was significantly larger in the SH rats [WKY = 0.73 ± 0.11 spikes/bin (*n* = 19) vs. 1.74 ± 0.32 spikes/bin (*n* = 20); **P = 0.009, *t*-test].

In both strains the MVC_like_ spike discharge showed respiratory modulation (Fig. 1, *A* and *B*). However, the average firing frequency of MVC_like_ SPN in SH rats was 58% higher than WKY [3.85 ± 0.39 Hz (*n* = 23) vs. 2.44 ± 0.35 Hz (*n* = 22); *P* = 0.011; Fig. 1*C*]. For each cell, action potential discharge was binned into eight 45° bins across the respiratory cycle (WKY *n* = 19, SH *n* = 20 SPN, activity averaged across 10 cycles for each cell). This respiratory phase analysis of firing showed that SPN in both strains had a peak of discharge in the late inspiratory (I) and early postinspiratory (PI) phases (Fig. 1, *D* and *E*; peak at 45° in both strains, one-way ANOVA) with a tendency for an earlier start to the inspiratory burst in the SH rat. Comparison of these firing histograms showed that both strain and the phase through the respiratory cycle were significant sources of variation [which show influence of both phase (*P* < 0.0001) and strain (*P* < 0.01) with an interaction *P* < 0.01, two-way mixed measures ANOVA] with a significant increase in the peak seen particularly in the 45° bin in the SH rat (*P* < 0.001, Bonferroni post hoc test). The increased overall firing rate of MVC_like_ SPN in SH rats comprised a potentiation of both the respiratory modulated component and a basal component (Fig. 1, *D* and *E*). The basal level of discharge was increased 1.87-fold in SH rats from 0.49 ± 0.08 spikes/respiratory cycle in WKY (*n* = 19) to 0.93 ± 0.15 spikes/respiratory cycle in SH rats (*n* = 20); *P* = 0.016. The amplitude of the peak discharge was also increased, from 0.87 ± 0.16 spikes/respiratory cycle (WKY, *n* = 19) to 1.96 ± 0.35 spikes/respiratory cycle (SH, *n* = 20; *P* < 0.0001; peak-to-peak). The degree of respiratory modulation of spike discharge, as measured by the peak-to-trough difference in spike count across the bins for each neuron, was amplified 2.2-fold in the SH rats [WKY 0.73 ± 0.11 spikes/respiratory cycle (*n* = 19) vs. SH 1.74 ± 0.32 spikes/respiratory cycle (*n* = 20); *P* = 0.002; Fig. 1*F*].

Importantly, in considering the origin of this altered respiratory modulation, we found no difference in respiratory rate (0.39 ± 0.04 WKY vs. 0.35 ± 0.03 Hz SH; *P* = 0.52) or inspiratory duration (539 ± 35 WKY vs. 466 ± 36 ms SH; *P* = 0.16; *n* = 31 WKY and *n* = 24 SH preparations). In both strains peripheral chemoreflex activation (50 ul, 0.03% NaCN; *n* = 21 WKY and *n* = 19 SH) produced similar increases in respiratory frequency (0.20 ± 0.03 Hz WKY vs. 0.16 ± 0.02 Hz SH; *P* = 0.39), increased phrenic amplitude (4.24 ± 0.87 μV WKY vs. 5.05 ± 1.42 μV SH; *P* = 0.73), and decreased heart rate (1.19 ± 0.11 Hz WKY vs. 0.98 ± 0.08 Hz SH; *P* = 0.12). However, peripheral chemoreflex activation produced a greater increase in firing in MVC_like_ SPN of SH than WKY rats [4.27 ± 0.81 Hz (*n* = 19) vs. 1.37 ± 0.31 Hz, (*n* = 21); *P* = 0.0018]. Given that respiratory and parasympathetic vagal measures of the magnitude of the chemoreflex are similar across the strains, this elevated firing response suggests altered excitability in the sympathetic vasomotor pathway in the SH rat downstream of the respiratory pattern generation network.

### Altered MVC_like_ Excitability in the SH Rat

**Fig. 2.**
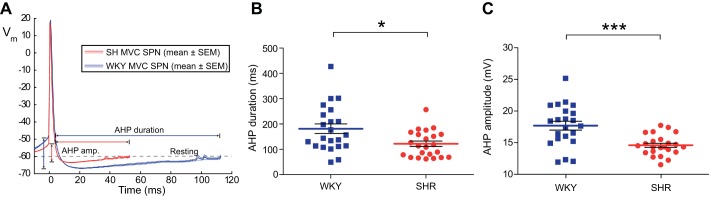
Reduction in afterhyperpolarization (AHP) of MVC_like_ SPN in SH rats. *A*: action potential waveforms from a representative WKY (blue) and SH (red) MVC_like_ SPN (average of 10 spikes ± SE) showing reduced AHP amplitude and duration. *B* and *C*: across the population the SH rat MVC_like_ SPN had shorter [120.8 ± 10.8 (*n* = 23) vs. 179.6 ± 19.5 ms (*n* = 22); *P = 0.01], smaller AHPs [14.6 ± 0.34 mV (*n* = 23) vs. 17.7 ± 0.71 mV (*n* = 22); ***P = 0.0002].

Given the increased firing activity of MVC_like_ SPN in the WHBP from the hypertensive strain we tested whether this was related to differences in their intrinsic electrophysiological properties? Comparison of MVC_like_ SPN between WKY and SH rats (Table 1) showed that they have similar resting membrane potentials and action potential threshold, amplitude and duration. Interestingly, given the increased ongoing firing frequency, the AHP was both smaller and shorter in the SH than the WKY (14.6 ± 0.3 vs. 17.7 ± 0.7 mV, *P* = 0.0002; 120.8 ± 10.8 vs. 179.6 ± 19.5 ms, *P* = 0.011; Fig. 2). In addition, the input resistance of MVC_like_ SPN was greater in the WKY [446 ± 51 MΩ (*n* = 18) vs. SH 307 ± 33 MΩ (*n* = 16); *P* = 0.033]. Thus the intrinsic properties of SPN differ between SH and WKY rats.

**Table 1.** Electrophysiological properties of MVC_like_ SPN in WKY and SH rats

Property	WKY (means ± SE)	WKY (*n*)	SH (means ± SE)	SH (*n*)	*P*
Frequency, Hz	2.44 ± 0.35	22	3.85 ± 0.39	23	0.01
Resting potential, mV	−53.0 ± 1.2	22	−51.5 ± 1.0	23	0.32
Input resistance, MΩ	446 ± 51	18	307 ± 33	16	0.03
Time constant, ms	28.4 ± 3.0	16	40 ± 6.7	15	0.12
Threshold, mV	−42.8 ± 1.0	22	−42.9 ± 1.0	23	0.97
Spike amplitude, mV	47.4 ± 2.1	22	47.6 ± 2.1	23	0.97
Spike duration, ms	3.40 ± 0.24	22	3.50 ± 0.22	23	0.99
AHP amplitude, mV	17.7 ± 0.71	22	14.6 ± 0.34	23	0.0002
AHP duration, ms	179.6 ± 19.5	22	120.8 ± 10.8	23	0.01

MVC_like_, muscle vasoconstrictor-like; SPN, sympathetic preganglionic neurons; SH, spontaneously hypertensive; WKY, Wistar-Kyoto; AHP, afterhyperpolarization.

### Enhanced Excitability and Output in the SH Is Specific to MVC_like_ SPN

To test whether these changes were generalizable to other classes of SPN we also analyzed data from cutaneous vasoconstrictor-like (CVC_like_) SPN as a comparator cell class that were characteristically inhibited by both peripheral chemoreflex and diving response (*n* = 15 WKY and *n* = 6 SH; Stalbovskiy et al. 2014). The CVC_like_ SPN showed no significant differences in their firing rate or intrinsic properties between SH and WKY rats (Table 2), suggesting that the changes in excitability are specific to the MVC class.

**Table 2.** Comparison of electrophysiological properties of CVC_like_ SPN across strains

Property	WKY (means ± SE)	WKY (*n*)	SH (means ± SE)	SH (*n*)	*P*
Frequency, Hz	1.45 ± 0.21	15	1.60 ± 0.61	6	0.73
Resting potential, mV	−51.81 ± 1.06	15	−54.30 ± 2.68	6	0.36
Input resistance, MΩ	304.9 ± 63.90	11	301.2 ± 44.60	5	0.65
Time constant, ms	26.60 ± 5.72	10	21.00 ± 6.66	5	0.69
Threshold, mV	−42.25 ± 0.95	15	−45.95 ± 2.52	6	0.17
Spike amplitude, mV	49.93 ± 2.75	15	51.00 ± 4.89	6	0.73
Spike duration, ms	3.28 ± 0.20	15	2.91 ± 0.58	6	0.31
AHP amplitude, mV	−18.56 ± 1.06	15	−17.33 ± 1.30	6	0.51
AHP duration, ms	297.1 ± 36.16	15	288.2 ± 99.71	6	0.37

CVC_like_, cutaneous vasoconstrictor-like.

### MVC_like_ SPN in the SH Rat Have Reduced Transient Rectification

**Fig. 3.**
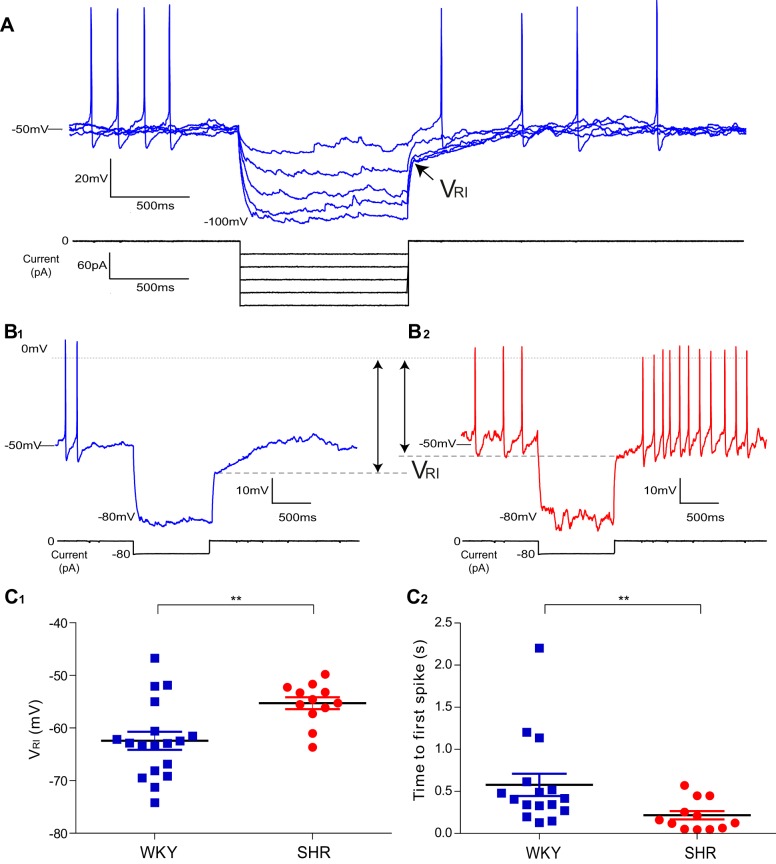
Diminished transient rectification in MVC_like_ SPN of SH rats. *A*: voltage responses of a MVC_like_ SPN of a WKY rat to family of hyperpolarizing current pulses. At the offset of hyperpolarizations greater than −75 mV, a clear repolarization inflection point was seen in the decay trajectory (arrow; *V*_RI_). This signaled the activation of the transient rectification (*I*_A_), which delayed repolarization and suppressed the firing activity of the cell. *B*: *V*_RI_ occurred at a more hyperpolarized level in WKY (*B*_*1*_), than the SH rat (*B*_*2*_). *C*_*1*_: grouped *V*_RI_ data (measured on repolarization from a potential of < −75 mV) showing it occurred at a more hyperpolarized level in WKY compared with SH rats [WKY = −62.4 ± 1.7 mV (*n* = 18); SH = −55.3 ± 1.1 mV (*n* = 12); *P* = 0.0043]. *C*_*2*_: time-to-first-spike (measured from the release of the hyperpolarizing current pulse) was shorter in the SH rat [WKY 577 ± 132 ms (*n* = 16) vs. SH 213 ± 53 ms (*n* = 12); **P < 0.0001].

We noted additional differences in the membrane potential responses of MVC_like_ SPN to current injection across strains that suggested an alteration in the transient rectification in the SH rat. Specifically, the recovery trajectory of the membrane potential from a hyperpolarized level (after current pulse injection) showed a clear inflection point on repolarization followed by a delayed return to rest in all of the WKY MVC_like_ SPN (*n* = 18; Fig. 3*A*). This inflection is a well-characterized consequence of activation of an A-current (*I*_A_) (Miyazaki et al. 1996; Pickering et al. 1991; Sah and McLachlan 1995; Whyment et al. 2011; Wilson et al. 2002; Zimmerman and Hochman 2010) and has been noted previously as a distinct feature of the MVC_like_ SPN (Stalbovskiy et al. 2014). The majority of MVC_like_ SPN in SH rats also exhibited an inflection (71%, 12/17), but it was markedly less pronounced than in the WKY (Fig. 3*B*). The remainder of SH MVC_like_ SPN (*n* = 5) did not exhibit any inflection on repolarization, instead showing a passive trajectory. The potential at which the inflection occurred (*V*_RI_) was measured at the abrupt transition from initial passive exponential repolarization to a shallower, almost linear d*V*_m_/d*t* (Fig. 3*A*). *V*_RI_ was shifted to a more depolarized potential in SH rats [WKY −62.4 ± 1.7 mV (*n* = 18) vs. SH -55.3 ± 1.1 mV (*n* = 12); *P* = 0.0043; Fig. 3*C*_*1*_]. As a consequence of this transient rectification the time-to-first-spike was much longer in WKY compared with SH rats [577 ± 132 ms (*n* = 16) vs. 213 ± 53 ms (*n* = 12); *P* = 0.0076; Fig. 3*C*_*2*_]. These data suggest there is a reduction in *I*_A_ in the MVC_like_ neurons of the SH rat.

### Modeling the Influence of the A-Current on SPN Excitability

**Fig. 4.**
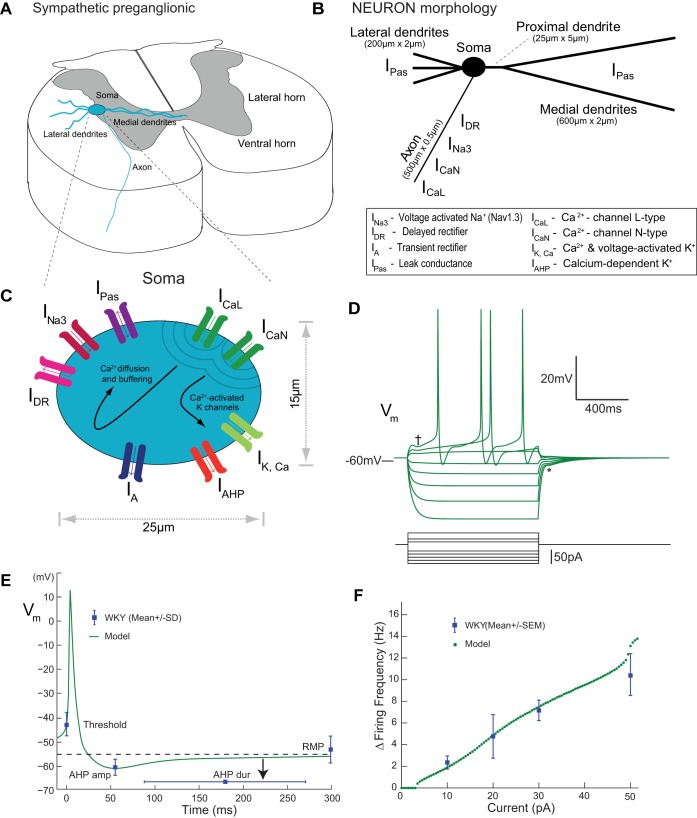
Model of an MVC_like_ SPN and comparison of electrophysiology to WKY data. *A*: schematic morphology of a SPN in the lateral horn of the spinal cord showing its position in the lateral horn, dendritic tree, and axon heading toward the ventral root. *B*: morphology of the SPN defined in NEURON with the distribution of ion conductances; the dendrites were passive and the axon had 4 voltage-dependent conductances (*I*_Na3_, *I*_DR_, *I*_CaL_, and *I*_CaN_). *C*: soma schematic showing conductances, intracellular buffers, and membrane mechanisms. *D*: membrane potential responses of the model to current pulse injections (1-s duration). Note the delay to firing (†) and the repolarization inflection (*V*_RI_) on return to rest after hyperpolarization (*). *E*: action potential firing was triggered in the model with a small current pulse (5 pA). The model spike threshold, AHP amplitude, AHP duration (arrow), and resting membrane potential (RMP) are all within a standard deviation of the experimental data for MVC_like_ SPN in WKY rats (full comparison in Table 3). *F*: firing frequency of the model to depolarizing current pulse injection fits the experimental data from MVC_like_ SPN in WKY 10 pA (2.4 ± 0.6 Hz; *n* = 19), 20 pA (4.8 ± 2 Hz; *n* = 5), 30 pA (7.2 ± 1 Hz; *n* = 20), and 50 pA (10.5 ± 10.9 Hz; *n* = 11); response to current pulse injection for 1 s.

Given the known influence of *I*_A_ on the excitability of other neurons (Connor and Stevens 1971; Rush and Rinzel 1995), we hypothesized that the alteration in transient rectification in MVC_like_ SPN of the SH rat may account for the altered repolarization and reduced size of AHP and the increased excitability. To test this hypothesis, we constructed a conductance-based compartmental model of the SPN in the WKY (see *Computational Overview*, Fig. 4, and appendix). We systematically adjusted the parameters of the model *I*_A_ to generate a biophysically accurate recapitulation of SPN transient rectifier kinetics in vitro (Bordey et al. 1995; Sah and McLachlan 1995; Whyment et al. 2011). The characteristics of the conductance closely matched the experimentally derived values (see appendix).

The electrophysiological characteristics of the SPN model both qualitatively and quantitatively resembled the experimental recordings of MVC_like_ SPN in WKY (Fig. 4 and Table 3). The model produced action potential firing after an initial delay in response to depolarizing current injection (Fig. 4*D*). It also shows an inflection and delayed return to rest on repolarization after the injection of a hyperpolarizing current pulse (Fig. 4*D*). The action potential waveform of our model cell was similar to the WKY MVC_like_ SPN with similar threshold, amplitude, duration, and AHP morphology (Fig. 4*E* and Table 3). Likewise the current-firing frequency relationship of the model was within the observed range for WKY MVC_like_ SPN (Fig. 4*F*).

**Table 3.** Electrophysiology of model SPN compared with WKY MVC_like_ SPN

Property	Model SPN	SPN In Situ (means ± SE)	Model WKY
Resting potential, mV	−55.0	−53.0 ± 1.2	2.0
Input resistance, MΩ	350	446 ± 51	96
Time constant, ms	41.5	28.4 ± 3.0	13.1
Threshold, mV	−39.1	−42.8 ± 1.0	3.7
Spike amplitude, mV	49.5	47.4 ± 2.1	2.1
Spike duration, ms	3.0	3.40 ± 0.24	0.4
AHP amplitude, mV	17.0	17.7 ± 0.71	0.7
AHP duration, ms	175	179.6 ± 19.5	4.6

### Relationship Between I_A_ and Model Excitability

**Fig. 5.**
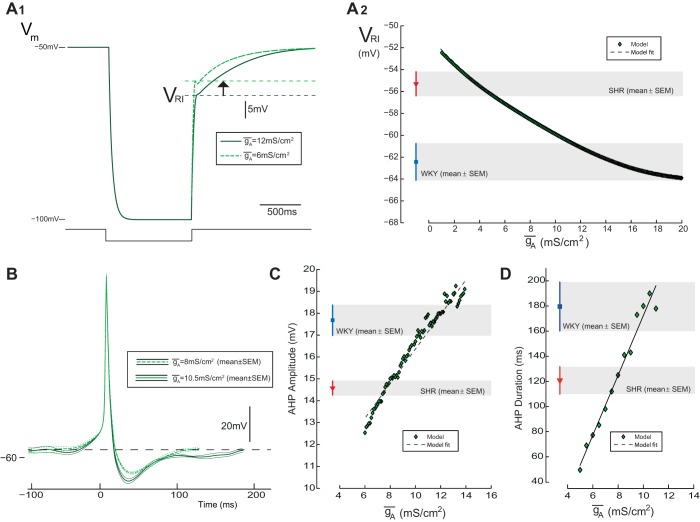
Influence of *I*_A_ on membrane excitability of the model. Reducing the maximum conductance density (*ḡ*_A_) alters the excitability of the model in a manner consistent with the SH data. *A*: step current pulses (1-s duration) were injected into the model cell from a potential of −50 mV, to hyperpolarize the cell to −100 mV to measure *V*_RI_. *A*_*1*_: as *ḡ*_A_ was reduced from 12 to 6 mS/cm^2^, *V*_RI_ shifted in a depolarizing direction (∼5 mV). *A*_*2*_: relationship between *ḡ*_A_ and *V*_RI_ showed that reduction of *ḡ*_A_ moved the level of *V*_RI_ from the WKY to SH range (shaded regions). *B*: AHP amplitude and duration in the model was measured from action potential waveforms generated from excitatory input [excitatory postsynaptic currents (EPSCs) recorded experimentally see Fig. 6]. Decreasing *ḡ*_A_ from 10.5 mS/cm^2^ reduced the AHP amplitude (*C*) and duration (*D*) from the range of WKY MVC_like_ SPN to values in the range seen in SH rats (shaded regions).

Having established that the model recapitulated many of the features of the WKY SPN we systematically examined how the A-current could influence excitability. We found that perturbation of the *I*_A_ conductance parameters [maximal conductance density *ḡ*_A_ and activation parameters *V*_½,*n*_ (half-activation voltage) and ζ_*n*_ (slope parameters) and equivalent inactivation parameters *V*_½,*l*_ and ζ_*l*_] were all able to alter the *V*_RI_, AHP amplitude, AHP duration, and firing frequency of the model. However, only *ḡ*_A_ altered those features in a manner consistent with the experimental data for MVC_like_ SPN in SH rats (Fig. 5). In contrast, the other A-current parameters that were tested exhibited contrary effects on the excitability and output of the model (see appendix). For example both of the inactivation parameters *V*_½,*l*_ and ζ_*l*_ had opposing effects on *V*_RI_ and firing frequency. The activation parameters (*V*_½,*n*_ and ζ_*n*_) both shifted *V*_RI_ in a depolarizing direction and increased the firing frequency; however, the AHP became larger (unlike that seen in the SH rat recordings).

**Fig. 6.**
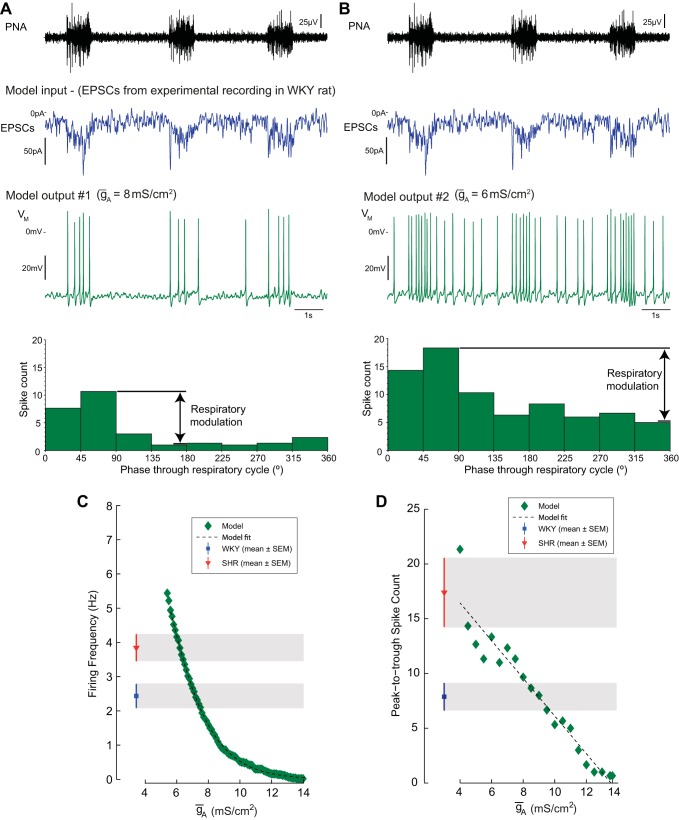
*I*_A_ shapes the output of the model. The model was challenged with a train of EPSCs [recorded in voltage-clamp (−53 mV) from a WKY rat MVC_like_ SPN over 100 s]. The output of the model (firing and pattern) was monitored as the *ḡ*_A_ was reduced. *A*: for high *ḡ*_A_ (8 mS/cm^2^), the model produced a low average firing frequency (1.8 Hz) with a degree of respiratory modulation (peak-to-trough = 10 spikes), consistent with the WKY data. *B*: as *ḡ*_A_ was reduced (to 6 mS/cm^2^), the average firing frequency (4.7 Hz) and degree of respiratory modulation (14 spikes) increased, as seen in the SH group. Graded reductions of *ḡ*_A_ produced a monotonic increase in the firing frequency (*C*) and respiratory-coupling (*D*) of the model into the ranges seen experimentally across the strains (shaded regions).

On this basis we focused on the influence of *ḡ*_A_ on cell excitability. Reduction of *ḡ*_A_ from 12 to 6 mS/cm^2^ caused a depolarizing shifted in *V*_RI_ of 5 mV (Fig. 5*A*_*1*_). A 74% reduction in *ḡ*_A_ recapitulated the experimentally observed difference in *V*_RI_ between strains (WKY −62.4 ± 1.7 mV to SH −55.3 ± 1.1 mV; Fig. 5*A*_*2*_). The action potential AHP morphology was also sensitive to variation of *ḡ*_A_ (Fig. 5*B*) with amplitude (Fig. 5*C*) and duration (Fig. 5*D*) decreased in proportion to *ḡ*_A_ [spike discharge driven in the model by excitatory postsynaptic current (EPSC) input shown in Fig. 6]. In response to a reduction in *ḡ*_A_ of 33.8 and 27.3%, the model AHP amplitude and duration (respectively) changed from the experimental WKY to the SH data (Fig. 5, *C* and *D*). Consistent with our experimental comparison of SPN from SH and WKY rats, these changes in *ḡ*_A_ had comparatively little influence on resting membrane potential and action potential threshold, amplitude or duration (Fig. 5*B*).

### Response of the Model SPN to Excitatory Postsynaptic Potential Is Enhanced by Reduction of ḡ_A_

To examine the influence of *I*_A_ on the pattern of firing activity we drove the model with experimentally recorded synaptic currents from a WKY MVC_like_ SPN (Fig. 6). Decreasing *ḡ*_A_ by 25% from 8 to 6 mS/cm^2^ increased the action potential response to the same synaptic drive by ∼3 Hz (Fig. 6, *A* and *B*, respectively). Examination of the *ḡ*_A_-firing frequency response curve showed that reducing *ḡ*_A_ by 12.7% (from 7.47 to 6.52 mS/cm^2^) was sufficient to increase the average firing frequency from the WKY to the SH values for MVC_like_ SPN (Fig. 6*C*). Thus relatively small alterations to *ḡ*_A_ can induce a marked increase in the firing frequency response to a given synaptic drive.

### Reducing ḡ_A_ Increases Sympathetic-Respiratory Coupling

We next queried whether such reductions *ḡ*_A_ could account for differences in sympathetic output across the respiratory cycle between strains (Fig. 6, *A* and *B*). Simulations with control and reduced *ḡ*_A_ both show a clear respiratory modulation of spiking reflecting the pattern of the underlying synaptic drive. The peaks in firing occurred during the I/PI phase with troughs during expiration. As *ḡ*_A_ was varied, the peak-to-trough difference in spike count followed a linear relationship (Fig. 6*D*), indicating that a reduction in the A-current density amplifies the degree of respiratory-sympathetic coupling in a manner consistent with the data for SH rats (cf Fig. 1).

**Fig. 7.**
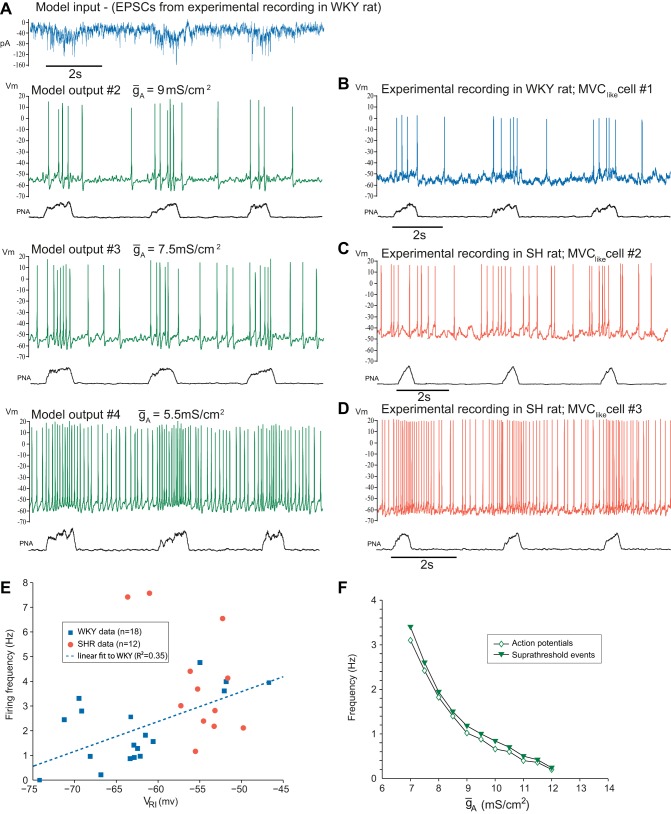
SPN output characteristic is reconfigured by *ḡ*_A._ *A*: pattern of discharge of the model, in response to a common EPSC drive recorded from a WKY MVC_like_ neuron (*top trace*), was closely comparable to that of experimentally recorded cells across strains. The frequency and respiratory modulation of firing increased in the model as *ḡ*_A_ was reduced (down the column). *B*–*D*: experimental recordings of MVC_like_ SPN. With higher values of *ḡ*_A_, the model exhibited strikingly similar discharge patterns to that seen in WKY MVC_like_ SPN (#1), whereas with low values of *ḡ*_A_ the model output more closely resembled recordings from SH rats (#2 and #3). *E*: firing frequency of recorded MVC_like_ SPN plotted as a function of *V*_RI_ for WKY (*n* = 18) and SH (*n* = 12). The WKY data were fit with a linear regression (*R*^2^ = 0.35) showing a positive correlation between *V*_RI_ and the spontaneous firing frequency. *F*: using the model we tested whether the effect of *ḡ*_A_ altered the frequency of threshold crossing synaptic events (generated from the EPSC train in *A*) by inactivating the sodium conductance to prevent action potential discharge. Comparison of the numbers of action potentials with the number of suprathreshold synaptic events revealed a close linkage across *ḡ*_A_ indicating that the major influence of the A conductance on discharge is through altered synaptic integration rather than by an action on the AHP. We also used the model to investigate the effect of altering *ḡ*_A_ on the number of underlying threshold crossing synaptic events (with sodium spiking inactivated) vs. the number of action potentials discharged to see whether its influence on firing frequency was via an action on synaptic integration or upon the after hyperpolarization (*F*). The event counts [excitatory postsynaptic potentials (EPSPs) and action potentials] under each condition follow a very similar relationship indicating that it is an increase in the number of threshold crossing events that drives the majority of the change in firing rather than a shortening of the refractory period after an action potential.

As *ḡ*_A_ is varied, the patterns of spike output from the model in response to the same experimentally recorded train of EPSCs closely resembled the patterns of activity of SPN from WKY and SH rats (Fig. 7, *A*–*D*). We sought evidence for this effect of the transient rectification in vivo by plotting *V*_RI_ (a measure of the strength of A-current) against the firing frequency for WKY (*n* = 18) and SH (*n* = 12) MVC_like_ SPN (Fig. 7*E*). This shows a continuum of values across strains with a trend towards higher firing frequencies with more depolarized values of *V*_RI_. This was clearly seen in the WKY population with a correlation between depolarized *V*_RI_ (less A-current) and firing frequency; the linear regression revealed that the strength of the transient rectification accounted for ∼35% of the variance in firing frequency seen across neurons.

### I_A_ Regulates Excitability by an Action on Synaptic Integration

To begin to explore the mechanism by which reduction in *I*_A_ increases action potential output we probed the influence of *ḡ*_A_ on the number of underlying threshold crossing synaptic events (with sodium spiking inactivated) compared with the number of action potentials discharged under control conditions (Fig. 7*F*). The event counts [suprathreshold excitatory postsynaptic potentials (EPSPs) and action potentials] under each condition follow a very similar relationship indicating that it is an increase in the number of threshold crossing events that drives the majority of the change in firing rather than a shortening of the refractory period after an action potential. Note also that changing *ḡ*_A_ had relatively little influence on the action potential threshold. These simulations suggested that the increase in output with reductions in *ḡ*_A_ is a consequence of altered synaptic integration; however, this did not preclude a possible difference in the synaptic drives across the strains.

### Synaptic Drive to MVC_like_ SPN in the SH and WKY Rats

**Fig. 8.**
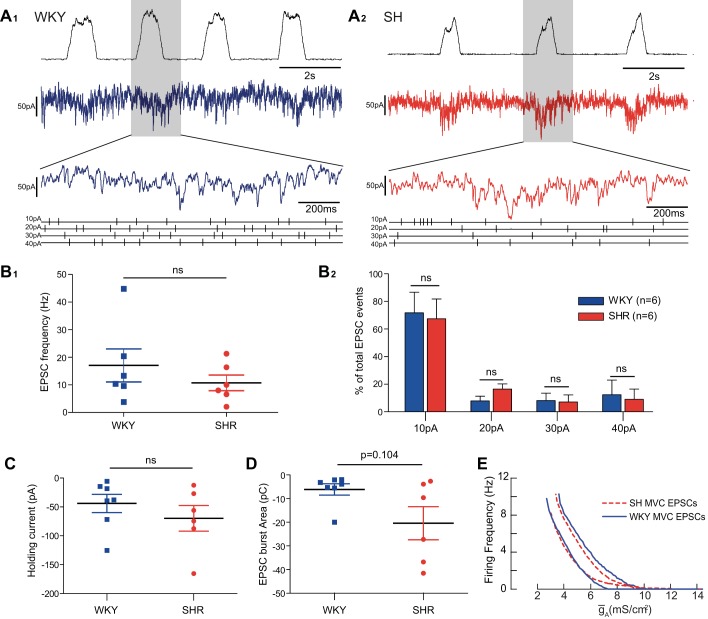
Synaptic input to MVC_like_ SPN in WKY and SH rats. MVC_like_ SPN in WKY (*n* = 7) and SH (*n* = 6) rats were voltage clamped close to rest (−53 mV) to record the spontaneously ongoing EPSCs. *A*: EPSCs incoming to an MVC_like_ SPN in a WKY (*A*_*1*_) and SH rat (*A*_*2*_). Over a 10-s period of recording, putative EPSCs were located using a peak-find algorithm (with a rise time of >1 ms and amplitude >10 pA in spike 2; *B*). The resultant output was manually validated against the raw data, and individual peaks were verified (shown expanded below with event trains). *B*_*1*_: frequency of incoming EPSCs of magnitude >10 pA was no different across the strains (WKY = 14.6 ± 3.4 Hz; SH = 12.7 ± 3.4 Hz; *P* = 0.71). *B*_*2*_: proportional amplitude distribution of incoming events was no different across the strains (data from recordings of 6 MVC_like_ SPN per strain, binned into 10- to 20-, 20- to 30-, 30- to 40-, or >40-pA events and expressed as a proportion of the total number of events over a 10-s period, two-way ANOVA with Bonferroni post hoc tests). *C*: mean holding current over the recording was no different across the strains (WKY = −49.6 ± 12.4 pA; SH = −56.6 ± 13 pA; *P* = 0.71). *D*: magnitude of the respiratory modulated burst of synaptics was quantified by integrating the current during inspiration. A greater synaptic charge transfer was apparent in the SH rat but did not reach significance (*P* = 0.104). *E*: model was challenged with EPSC traces taken from each strain [50-s duration; 2 WKY (blue), 2 SH (red dashed)]. The output discharge of the model, and its relationship to *ḡ*_A_, was seen to be relatively independent to the strain of origin of the EPSCs.

We analyzed the properties of the synaptic input to the MVC_like_ SPN to see whether the strains had different synaptic drives. The MVC_like_ SPN (*n* = 7 WKY and *n* = 6 SH) were voltage clamped close to rest (*V*_h_ = −53 mV) to obtain a measure of the frequency, amplitude, and respiratory modulation of incoming EPSCs (Fig. 8). This synaptic drive displayed respiratory modulation in both strains (Fig. 8, *A*_*1*_ and *A*_*2*_), with the larger amplitude events (>30 pA) clustered during the I and PI phases. The mean holding current was not different across the strains (WKY −49.6 ± 12.4 pA vs. SH −56.6 ± 13 pA; *P* = 0.71; Fig. 8*C*). Similarly, there was no difference in the frequency of occurrence of the synaptic events (>10 pA; Fig. 8*B*_*1*_) across the strains (WKY 14.6 ± 3.4 Hz, compared with 12.7 ± 3.4 Hz in SH rats; *P* = 0.71). There was no difference in the proportional distribution of synaptic amplitudes across the strains (Fig. 8*B*_*2*_, two-way ANOVA, Bonferroni post hoc tests). We also made an assessment of the respiratory-modulated component of the synaptic drive. The synaptic charge transfer (measured as the area under the inspiratory burst) of the respiratory modulated component tended to be smaller in WKY (−6.0 ± 2.4 vs. −20.3 ± 7.0 pC; *P* = 0.104; Fig. 8*D*). Therefore, based on this sample of voltage-clamp recordings, we were unable to demonstrate significant differences in the synaptic input to MVC_like_ SPN across the strains of rat despite there being clear difference in firing frequency under current-clamp conditions.

### Influence of ḡ_A_ on Model Response to EPSCs from MVC_like_ SPN Across Strains

Given that there was a trend towards altered respiratory modulation of the synaptic drive to the MVC SPN in the SH rat we examined the influence of *ḡ*_A_ on model SPN firing frequency (Fig. 8*E*) when driven with synaptic inputs from MVC_like_ SPN from WKY and SH rats (*n* = 2 each strain, at each end of the range of amplitudes). The average firing frequency produced by the model in response to these inputs, and its dependence on *ḡ*_A_, followed a similar profile, irrespective of the source of the input across the SH and WKY strains (Fig. 8*E*).

### I_A_ Regulates the Decay of EPSPs (and Hence Summation) in SPN

**Fig. 9.**
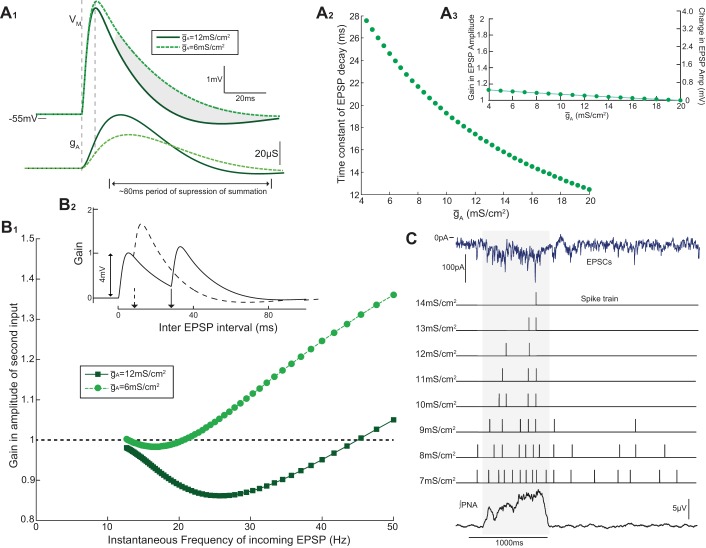
*I*_A_ functions as a low-pass filter of incoming EPSPs. The influence of *I*_A_ on synaptic integration in the model was investigated. *A*: injecting a point synaptic conductance (a double-exponential with rise time of 1.5 ms and decay time of 2 ms) produced an EPSP with a profile typical of those recorded in SPN (Spanswick et al. 1998). As was reduced from 12 to 6 mS/cm^2^, the time constant of decay increased considerably (*A*_*2*_), whereas EPSP amplitude increased only marginally (<5%; *A*_*3*_); this effect is explained by the relatively slow activation of the A conductance as indicated beneath where the peak conductance change occurs after the peak of the EPSP. *B*: to assess the influence of *ḡ*_A_ on synaptic summation 2 EPSPs were delivered at varying intervals (*B*_*2*_). For *ḡ*_A_ = 6 mS/cm^2^, the gain of the 2nd pulse (as a ratio of the single pulse height) increased with frequency (*B*_*1*_). For *ḡ*_A_ = 12 mS/cm^2^, gain was suppressed (i.e., <1) at high frequencies (10–45 Hz) and was unitary at low frequencies (<10 Hz). This suppression of gain is due to the influence of *I*_A_ on the decay of the EPSP. *C*: an experimentally recorded EPSC train (1 respiratory cycle) was injected into the model, and the output generated was converted into a spike train. For high values of *ḡ*_A_, the model filters out the synaptics incoming at high-frequency during the inspiratory phase. As *ḡ*_A_ was reduced, the model transforms more of the high-frequency events during the I phase into action potentials, and the respiratory-modulated burst increases in amplitude and starts earlier in the cycle.

How do variations in *ḡ*_A_ lead to differences in synaptic integration and output of the model? To address this question we examined the influence of *I*_A_ on the subthreshold summation of EPSPs (Fig. 9). The model SPN was challenged with a synthetic synaptic input to mimic a typical EPSP in SPN (Spanswick et al. 1998). The rate of decay of the resultant EPSP increased with *ḡ*_A_ (with a time constant of 25 ms at 6 mS/cm^2^ to 17.5 ms at 12 mS/cm^2^; Fig. 9*A*_*2*_), but the EPSP amplitude was only minimally affected (<5% over the same range of *ḡ*_A_; Fig. 9*A*_*3*_). To explore the influence of this change in EPSP decay on summation, we generated pairs of identical EPSCs that were played into the model at varying intervals across a range of *ḡ*_A_ values (Fig. 9*B*_*1*_; high *ḡ*_A_: 12 mS/cm^2^ and low *ḡ*_A_: 6 mS/cm^2^). The gain index was measured as the summated amplitude of the second pulse, as a ratio of the single pulse height (Fig. 9*B*_*2*_). This showed that *I*_A_ acts to decrease the summation of EPSPs incoming in the frequency range between 15 and 40 Hz (Fig. 9*B*), effectively imposing a low-pass filter characteristic on the SPN output.

We examined how this influence of *ḡ*_A_ on synaptic integration could shape the sympathetic output using a more physiological input of the experimentally recorded EPSC train over a respiratory cycle (Fig. 9*C*). The model generated progressively more action potentials from the high-frequency synaptic events incoming during the I/PI phase as *ḡ*_A_ was reduced and the respiratory-modulated burst emerged earlier in the I phase. This low-pass filtering property of the A-current therefore influences the pattern of firing of MVC_like_ SPN by attenuating summation particularly during the respiratory barrage of synaptic inputs. Diminution of *I*_A_, as seen in the SH rat, thus has a profound effect on the spiking output through a failure of the low-pass filtering action on the incoming synaptic drive.

### Blocking I_A_ with Intrathecal 4-AP Increases SNA and Traube-Hering Wave Amplitude

**Fig. 10.**
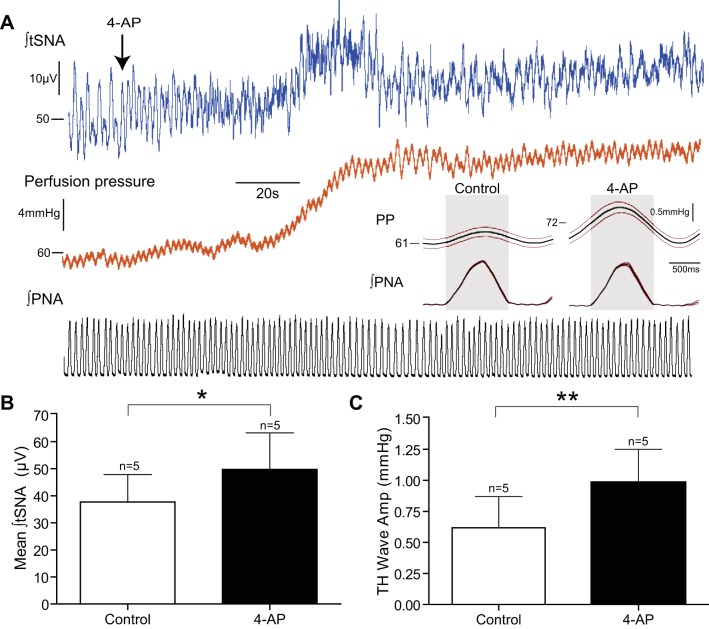
Intrathecal 4-aminopyridine (4-AP) increases thoracic sympathetic nerve activity (tSNA) in WKY rats. Recordings of tSNA alongside phrenic and perfusion pressure were made from DAPR of WKY rats (*n* = 5). *A*: intrathecal 4-AP (100-mM bolus) increased tSNA by blocking spinal *I*_A_. The perfusion pressure was increased due to the increased sympathetic outflow causing vasoconstriction. In addition the respiratory related fluctuation in perfusion pressure (Traube-Hering wave) was also increased in amplitude, as shown in the PNA-triggered average waveforms *insets*. *B*: mean tSNA in control conditions (37.7 ± 10.2 μV) increased (49.7 ± 13.5 μV; *P = 0.03) following 4-AP administration. This highlights the importance of the A-current in regulating spinal sympathetic tone. *C*: Traube-Hering wave amplitude (0.37 ± 0.15 mmHg) also increased (0.99 ± 0.26 mmHg; **P < 0.01), suggesting amplified respiratory-sympathetic coupling.

To test the principle that the A-current is acting to filter and regulate the sympathetic outflow we recorded thoracic SNA in DAPR of WKY rats (*n* = 5) and examined the response to an intrathecal bolus (100 nM in 2–10 ul) of the potassium channel blocker 4-AP (Fig. 10). Both SNA and perfusion pressure increased in response to the bolus (Fig. 10*A*). Thoracic SNA was significantly increased by 4-AP (Fig. 10*B*; baseline, 37.7 ± 10.2 μV; 4-AP, 49.7 ± 13.5 μV; *P* = 0.03). The amplitude of Traube-Hering waves, measured as the peak-to-trough change in perfusion pressure, was more than doubled after the application of 4-AP (Fig. 10*C*; baseline 0.37 ± 0.15 vs. 4-AP 0.99 ± 0.26 mmHg; *P* = 0.01). These findings are consistent with the A-current playing a role in governing the sympathetic outflow at a spinal level and its blockade increases both the sympathetic outflow and its consequent vasoconstrictor action on the vasculature.

## DISCUSSION

In this investigation we obtained whole cell recordings from MVC_like_ SPN in SH and WKY rats in situ to identify differences in the integrative properties and synaptic drive in the hypertensive strain that are present before the development of overt hypertension. We find that the SH rat MVC_like_ SPN have a 1.6-fold higher frequency of action potential discharge with a greater degree of respiratory modulation of their firing than MVC_like_ SPN in WKY rats (no difference across strains was noted in the CVC_like_ SPN in any parameter). This increase in SH MVC_like_ discharge was associated with a smaller and shorter AHP accompanied by signs of a weaker transient rectification.

This led us to examine the hypothesis that the increased firing in MVC_like_ SPN in the SH rat was due to reduced *I*_A_. We explored this hypothesis by constructing a mathematical model of MVC_like_ SPN in the NEURON environment (Hines et al. 2004) with a high-fidelity reconstruction of *I*_A_ based on experimentally derived values (Bordey et al. 1995; Sah and McLachlan 1995; Whyment et al. 2011). Besides recapitulating many of the characteristic intrinsic features of SPN noted from experimental studies, this model produced the anticipated patterns of action potential discharge when challenged with synaptic drives recorded from SPN in situ. The model was found to be particularly sensitive to variation of the maximal conductance density (*ḡ*_A_); for example, a 12.7% reduction could change the action potential discharge frequency of the model from a WKY to a SH rat characteristic and also increased the model SPN intrinsic excitability in a manner consistent with that seen experimentally. On the basis of this modeling data we propose that the pattern of increased sympathetic activity in the SH rat could be adequately explained by the reduction in *I*_A_. Consistent with this proposition we found that intrathecal administration of 4-AP in the DAPR preparation (Pickering et al. 2006; Sadanada et al. 2011), to block the *I*_A_ at a spinal level, produced a striking increase in SNA, accompanied by increased vascular resistance and greater amplitude of Traube-Hering waves, alterations similar to those reported for the SH rat at this age (Simms et al. 2009). Given that these changes in *I*_A_ predate the overt development of hypertension in the SH rat we speculate that they may be causal to, rather than consequential upon, the development of hypertension.

### Characteristics of MVC_like_ SPN in SH Rats

The MVC_like_ SPN of WKY rats exhibited characteristic electrophysiological properties, similar to those reported in vitro (Sah and McLachlan 1995; Spanswick and Logan 1990; Wilson et al. 2002; Yoshimura et al. 1986b) and in situ (Stalbovskiy et al. 2014) and in vivo (Dembowsky et al. 1986). The MVC_like_ SPN in the SH strain were recognizably similar to those previously documented in Wistar rats in that they showed a respiratory modulated pattern of ongoing action potential discharge driven by underlying EPSPs (Stalbovskiy et al. 2014). There was increased firing in the SH rat strain and enhanced respiratory coupling, mirroring whole nerve recordings (Simms et al. 2009, 2010) and reinforcing the principle that there are central changes in the processing of the sympathetic outflow in the SH rat (Morrison and Whitehorn 1984; Schramm and Barton 1979; Schramm et al. 1979).

The resting membrane potential in MVC_like_ SPN in the SH rat was not different from that in the WKY. Similarly, there was no change in the threshold for action potential discharge or in the spike amplitude, suggesting that the altered excitability was unlikely to be due to alterations in the sodium or calcium conductances. However, we noted that the SH rat had smaller AHPs and also exhibited a depolarizing shift in the repolarization inflection point (*V*_RI_) followed by an accelerated repolarization to rest. These latter two features are considered to be characteristics of the transient rectification in SPN (Bordey et al. 1995; Dembowsky et al. 1986; Miyazaki et al. 1996; Pickering et al. 1991; Sah and McLachlan 1995; Whyment et al. 2011) and suggested that there may be an alteration in its expression or kinetics in the hypertensive strain. Intriguingly, we noted a positive correlation between the *V*_RI_ and the baseline firing frequency of the MVC_like_ SPN, suggesting that it may play a role in determining the excitability and output of these neurons in situ.

### Influence of I_A_ on MVC_like_ SPN Model Excitability

Independent variation of *I*_A_ parameters could markedly alter the excitability of the SPN model. Reducing *ḡ*_A_ shifted *V*_RI_ in a depolarizing direction from a WKY range to that seen in the SH rat and also decreased AHP amplitude and duration, as seen in the SH rat. A similar action has been shown experimentally in SPN in vitro where blockade of *I*_A_ with 4-AP was seen to markedly reduce AHP duration and amplitude (Wilson et al. 2002). The parameters describing the steady-state kinetics of the conductance were also systematically investigated [including the activation parameters (*V*_½,*n*_ and ζ_*n*_) and inactivation parameters (*V*_½,*l*_ and ζ_*l*_)] to see if they could recapitulate the SH data. Although these parameters all influenced model excitability, they each produced contrary changes in either *V*_RI_ or in AHP amplitude and duration. Therefore, we parsimoniously identified *ḡ*_A_ as being the best candidate parameter and used it to probe the influence of *I*_A_ on SPN excitability.

### I_A_ Sculpts SPN Responses to Incoming Synaptic Drives

When challenged with experimentally recorded EPSP trains, a reduction of *ḡ*_A_ increased the action potential output of the SPN model to a range seen in the experimental recordings from SH rats and similarly increased the degree of respiratory coupling. This reduction in *ḡ*_A_ in the SPN model was therefore sufficient to recapitulate the altered pattern of output and respiratory modulation of MVC_like_ SPN in SH rats. We also noted that reduction of *ḡ*_A_ shifted the phase of the start of the respiratory-related burst of firing to occur earlier in inspiration, accounting for a phenomenon noted in previous studies of sympathetic-respiratory coupling recorded from whole nerves in the SH rat (Czyzyk-Krzeska and Trzebski 1990; Simms et al. 2009, 2010).

### I_A_ Tunes the Gain of Synaptic Integration

We used the model to gain insight into the mechanics of how the A-current could be acting to alter SPN integration and excitability. The A-current in SPN is unusual in that it has a both relatively slow activation and very slow inactivation (in comparison to that found in many mammalian CNS neurons; Jerng et al. 2004), resulting in a hyperpolarizing current that lasts for many hundreds of milliseconds (Whyment et al. 2011). This prolonged duration makes it particularly suited to influence events in a frequency range that is associated with the respiratory modulation of SPN activity. Our in silico experiments reveal that this long-lasting *I*_A_ endows SPN with the ability to apply a low-pass filter to barrages of inputs, with high levels of *ḡ*_A_ allowing only sparse generation of action potentials with each respiratory cycle. As the density of *I*_A_ is reduced, each high-frequency synaptic barrage produces a larger burst of action potentials.

The modeling also reveals that *I*_A_ decreases the decay time constant of EPSPs, without substantial effects on the magnitude of the EPSP (because of its slow activation characteristic). This suppresses high-frequency (>12 Hz) summation of EPSP inputs, hence allowing the neuron to only respond to the strongest EPSP trains incoming during respiratory modulation, thus acting as a low-pass filter. A previous experimental and in silico study of sympathetic postganglionic neurons reached a similar conclusion showing that *I*_A_ also acted to speed the decay of nicotinic EPSPs, making summation less likely (Cassell and McLachlan 1986), but it is clear that this role is likely to be even more important in the preganglionic neuron that is actively integrating high-frequency synaptic barrages (Stalbovskiy et al. 2014). We hypothesize that this low-pass filtering property is attenuated in MVC_like_ SPN of the SH rat, resulting in the increased summation of synaptic drives and thus greater transmission of high-frequency, respiratory-modulated bursting to the vasculature.

### Is the Excitatory Synaptic Drive Altered in the SH Rat?

An alternative and/or additional mechanism for the alteration in firing frequency of SPN in the SH rat would be through a change in the synaptic drive to the MVC_like_ SPN (Sved et al. 2003). To our knowledge no intracellular recordings of SPN have been made to date in the hypertensive strain and so our recordings provide a first direct measure of the synaptic drive. The voltage-clamp recordings obtained from the MVC_like_ SPN showed common patterns of input across strains, with a trend towards an elevation in the respiratory-coupled excitatory drive in SH rats, but we found no evidence for a change in the basal rate or amplitude of synaptic events. We also observed that playing these synaptic current traces into the SPN model recapitulated the patterns of action potential discharge (so we have some confidence in their fidelity) and in each case the resulting output was quantitatively sensitive to the maximal current density of *I*_A_. It should be noted, however, that it is challenging to analyze such massed activity into the component synaptic events and we are only able to resolve the larger events above the baseline; therefore, there are limits to our ability to discern specific drives. Hence, we cannot discount the possibility of an altered descending drive from the brainstem as has been suggested by the recent findings of increased respiratory drive to presympathetic neurons in the SH rat (Moraes et al. 2014). Further studies to selectively manipulate the descending drives to SPN (e.g., Abbott et al. 2009) or the use of focal application of excitatory amino acid antagonists (Stalbovskiy et al. 2014) will be required to help resolve this question of whether there is altered strength of specific descending drives.

### Loss of Transient Rectification in MVC_like_ SPN in SH Rats

Studies of splanchnic (Morrison and Whitehorn 1984) and renal/adrenal (Schramm and Chornoboy 1982) sympathetic outflows in SH rats have attributed the increased activity of the sympathetic pathway to changes at a central and indeed spinal level, respectively. The impact of spinal cord processes upon the response to descending drives has been elegantly demonstrated in an optogenetic stimulation study of RVLM C1 neurons that showed a striking attenuation of the sympathetic response to this descending drive when it was repeated at short intervals (<2 s) (Abbott et al. 2009). This potent filtering effect (previously referred to as the “silent period”) was attributed to the intrinsic properties of the SPN and places a restriction on the magnitude of the response that can be obtained from a brainstem input. Interestingly, decreases in the sympathetic silent period have been reported in young, prehypertensive SH rats, suggesting that the altered excitability may have its origins in changes to the rectifying properties of SPN (Schramm and Barton 1979). Our in situ and in silico findings provide a potential explanation for these experimental observations; the increased intrinsic excitability, recorded in situ and recapitulated in silico by reducing *ḡ*_A_, is equivalent to the reduced silent period in the SPN. An attenuation of *I*_A_ could, therefore, underlie the previously reported hyperresponsiveness (Schramm and Barton 1979).

### Regulation of Transient Rectification in MVC_like_ SPN in SH Rats

Given that there is an underlying heritable basis to the generation of hypertension in the SH strain (albeit with a genetic complexity; Marques et al. 2010), it is interesting to consider whether a mutation in one of the A-current subunits or the regulatory proteins could underpin the pathology. Such channelopathies underpin a range of neurological disorders (Kullmann 2010), although to date there are relatively few reports of syndromes consequent upon loss of the potassium channel genes responsible for the A-current. There has been a single report of temporal lobe epilepsy associated with a mutation of the K_V_4.2 (Singh et al. 2006), but knockout studies have suggested that this produces a modest change in seizure threshold and cardiac investigations in the same mouse line showed that the phenotype is relatively benign with no overt cardiovascular pathology perhaps because of compensation from other potassium channel subunits (Guo et al. 2005). It should also be noted that the studies of Whyment et al. (2011) have suggested that the SPN transient rectification is likely mediated by Kv4.1 and Kv4.3 so we may not expect to find a sympathetic phenotype in the Kv4.2 knockout.

In this context it may be significant that alteration in excitability seen in our study was restricted to MVC_like_ SPN and did not extend to changes in CVC_like_ neuronal activity, implying a functional, cell-type selectivity in the deficit, rather than a global phenotype. While this change in the A-current in the SH rat could still be a manifestation of cell class restricted inherited predisposition or susceptibility (i.e., affecting MVC_like_ but not CVC_like_) on the basis of specific genetic expression profiles, it could also be a consequence of a targeted signaling event results from differential modulation of MVC_like_ and CVC_like_ neurones. There are precedents for such induced changes in the long-term regulation of *I*_A_; for example, in induced temporal lobe epilepsy models there is an increase in excitability due to reductions in the A-current (Bernard et al. 2004). Similarly, β-adrenoceptor-mediated elevations of cAMP and activation of downstream kinases have been shown to produce a depolarizing shift in the activation potential of *I*_A_ in hippocampal CA1 neurons leading to an increase in neuronal excitability (Hoffman and Johnston 1998). The SH rat has been shown to have altered noradrenergic neuronal function in the brainstem, which may lead to altered norepineprine release in the spinal cord (Kasparov and Teschemacher 2008), which could provide a mechanism for descending neuromodulatory regulation of *I*_A_ (cf. Hoffman and Johnston 1998). This may be relevant to the recently reported increase in sensitivity of the peripheral chemoreflex (McBryde et al. 2013; Moraes et al. 2014), which is proposed to be driving an increase in sympathetic outflow as this could be acting via altered catecholaminergic (or other neuromodulator) signaling to the cord from the RVLM.

Reductions in *I*_A_ in presympathetic cardiovascular control centers have been reported in induced (rather than genetic) models of hypertension (Belugin and Mifflin 2005; Sonner et al. 2008), and these changes have been proposed to contribute to the hyperexcitability. Alterations in *I*_A_ have also been reported in sympathetic postganglionic neurons from SH rats that show primary increases in the degree of inactivation in the SH strain (increasing excitability), but these are also accompanied by compensatory increases in the maximal conductance density *ḡ*_A_ (Robertson and Schofield 1999), which are consequent upon the development of hypertension. These studies all support the principle that the A-current can be altered in central cardiovascular control circuits in models of hypertension.

### Importance of Altered Bursting Activity of MVC_like_ SPN

Previous studies of sympathetic stimulation indicated that grouped stimuli could induce greater contractile responses in mesenteric arteries (Nilsson et al. 1985), suggesting that bursting preferentially regulates vascular resistance. Recent findings in humans have shown that vascular conductance responds to bursting SNA and that the vasoconstriction is dependent on burst amplitude and patterning (Fairfax et al. 2013). On this basis we anticipate that the amplification of the respiratory component of MVC_like_ SPN activity in the SH rat by a loss of *I*_A_ would therefore be expected to increase vascular resistance in excess of that predicted by simple consideration of the increase in tonic firing rate. It should also be noted that recent in silico investigations have shown that populations of neurons transform common inputs to synchronous output with greater fidelity at lower values of *ḡ*_A_ (Barreiro et al. 2012). Thus the reduced *ḡ*_A_ in SH rats would be expected to produce an enhanced synchrony of respiratory-modulated bursts across the population of MVC_like_ SPN. The resultant synchrony and amplification of norepineprine release onto the artery wall would be expected to also increase the respiratory modulated vasoconstriction (i.e., Traube-Hering waves) and blood pressure in the SH rat. Our experimental finding that spinal administration of 4-AP to the WKY rat produces just such a pattern of effects supports this contention notwithstanding the effects that the antagonist will have had upon other spinal circuits.

### Concluding Remarks

Our in situ and in silico experiments indicate that the transient rectification of SPN plays a key role in the process of sympathetic integration, acting as a potentially tunable low-pass filter whose slow kinetics are suited to regulation of the amplitude of bursting discharges. We provide evidence that dysfunction of this filter may be sufficient to recapitulate the experimental findings from the SH rat, and although this finding does not preclude altered brainstem mechanisms, it does highlight the importance of SPN properties in contributing to the elevated SNA in the prehypertensive rat. These changes in MVC_like_ SPN properties are found before the onset of hypertension (but at a time when the vascular resistance is already beginning to increase; Simms et al. 2009) and therefore may be causal rather than consequential. These conclusions highlight the key importance of the intrinsic properties of the SPN in shaping the sympathetic output to the vasculature in pathological conditions and identify it as a possible locus for intervention.

## GRANTS

This study was supported by the British Heart Foundation (Grant PG/06/084, PI: J. F. R. Paton) and also by The Wellcome Trust (Grant 088373, PI: A. E. Pickering). L. J. B. Briant is supported by a Biotechnology and Biological Sciences Research Council/Engineering and Physical Sciences Research Council PhD Studentship. A. E. Pickering is a Wellcome Trust Senior Clinical Research fellow.

## DISCLOSURES

No conflicts of interest, financial or otherwise, are declared by the author(s).

## AUTHOR CONTRIBUTIONS

Author contributions: L.J.B.B., A.O.S., M.F.N., A.R.C., and A.E.P. conception and design of research; L.J.B.B., A.O.S., and A.E.P. performed experiments; L.J.B.B., A.O.S., A.R.C., and A.E.P. analyzed data; L.J.B.B., A.O.S., M.F.N., A.R.C., and A.E.P. interpreted results of experiments; L.J.B.B., A.O.S., and A.E.P. prepared figures; L.J.B.B., A.R.C., and A.E.P. drafted manuscript; L.J.B.B., A.O.S., M.F.N., A.R.C., and A.E.P. edited and revised manuscript; L.J.B.B., A.O.S., M.F.N., A.R.C., and A.E.P. approved final version of manuscript.

## Figures and Tables

**Fig. A1.**
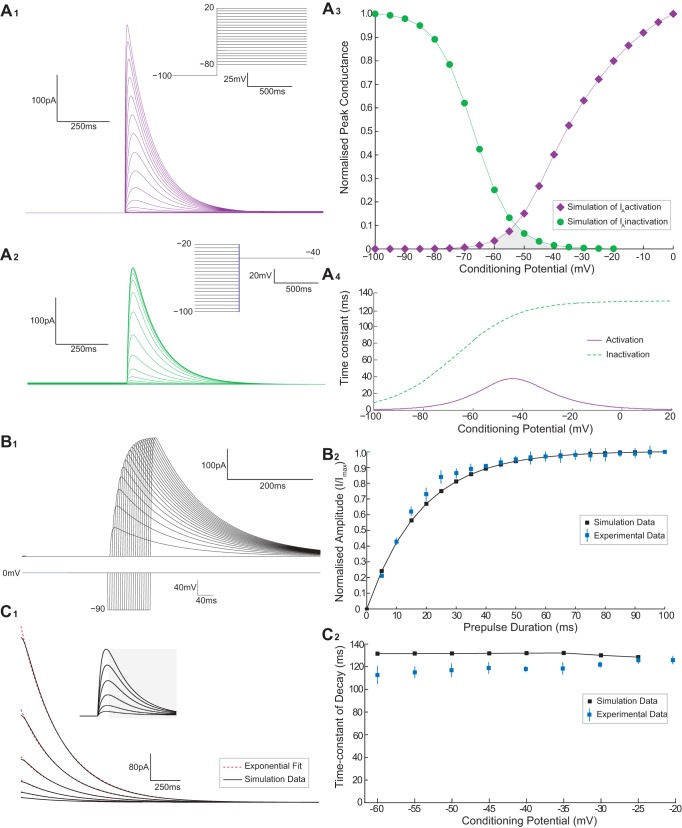
Comparison of model A-current kinetics with experimental data. *A*_*1*_: voltage-dependent activation investigated by applying depolarizing voltage steps from a holding potential of −100 mV. *A*_*2*_: steady-state inactivation was investigated with a voltage-step command to −40 mV applied from holding potentials of −100 to −20 mV. *A*_*3*_: plot of the activation (diamonds) and steady-state inactivation (circles) of *I*_A_, expressed as the normalized peak conductance, as a function of the conditioning potential. Note the shaded window current with amplitude 0.1 open probability. *A*_*4*_: plot of the time constants of activation and inactivation in the model as a function of conditioning potential. *B*_*1*_: rate of recovery from inactivation of *I*_A_ was tested by holding at zero to completely inactivate, then applying a hyperpolarizing voltage prepulse to −90 mV of varying duration (2–100 ms) before stepping back to 0 mV. *B*_*2*_: normalized peak conductances (black squares), plotted as a function of recovery interval, fit the experimental data (blue squares). *C*: decay time course of model *I*_A_ fits the experimental data. *C*_*1*_: same conductance traces as shown in *A*_*2*_ with single exponential fits (dashed lines) to the decay profile (*R*^2^ > 0.95). *C*_*2*_: decay time constant of these single exponential fits, plotted as a function of the conditioning potential. Note the difference between the time constants in the model (black squares) and experimental data (blue squares) is small (≤15%). [Experimental data replotted with permission from Whyment et al. (2011) (Fig. 2) and are expressed as means ± SE.]

**Fig. A2.**
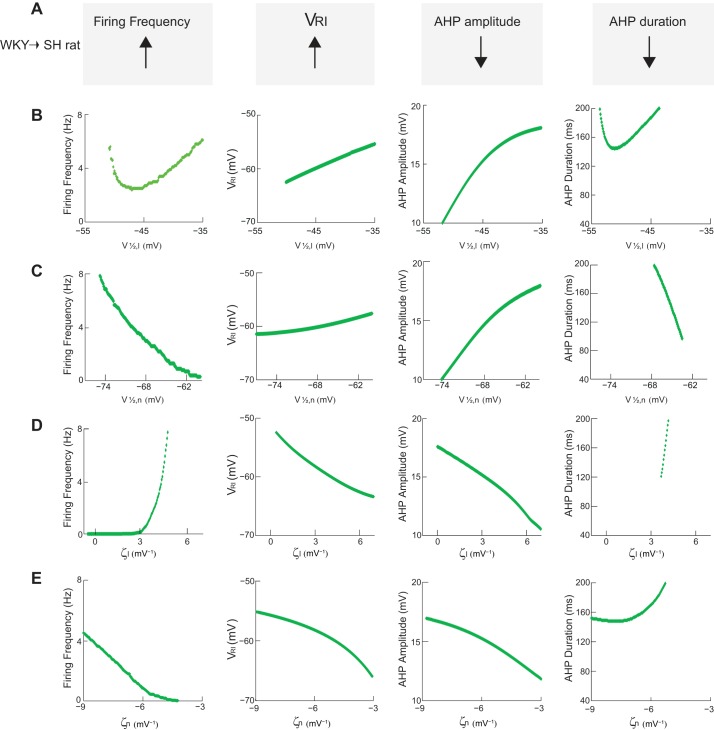
Sensitivity of model to *I*_A_ parameters. *A*: summary of experimental data: MVC_like_ SPN in the SH rat exhibited increased firing frequency, a more depolarized repolarization inflection point (*V*_RI_) and decreased AHP amplitude and duration. We sought *I*_A_ parameters that could recapitulate these features. *B*–*E*: model output (firing frequency) as a function of *I*_A_ steady-state parameters was investigated by injecting a train of experimentally recorded EPSCs from a WKY rat (Fig. 6). AHP morphology was measured following a brief (0.5 ms) large amplitude (2 nA) current pulse. Altering *V*_½,*l*_ (*B*), *V*_½,*n*_ (*C*), ζ_*l*_ (*D*), and ζ_*l*_ (*E*) could not parsimoniously reproduce the pattern of altered SPN excitability recorded across the strains.

## Glossary

ḡ_A_: *Maximal conductance density of* I_*A*_ (*mS/cm^2^*)
k_n_: *Activation slope factor of* I_*A*_ (*mV^−1^*)
k_l_: *Inactivation slope factor of* I_*A*_ (*mV^−1^*)
V_*m*_: *Membrane potential (mV)*
V_*RI*_: *Repolarization inflection potential (mV)*
V_½,n_: *Half-activation of* I_*A*_ (*mV*)
V_½,l_: *Half-inactivation of* I_*A*_ (*mV*)
ζ_n_: *Valence of activation gate of* I_*A*_ (*mV^−1^*)
ζ_l_: *Valence of inactivation gate of* I_*A*_ (*mV^−1^*)

## Appendix

### Model Equations

A compartmental model of a MVC_like_ SPN was constructed within the simulation environment NEURON v7.3 (Carnevale and Hines 2006). Code for the model has been deposited on ModelDB (Hines et al. 2004) and has ModelDB Accession No. 151482. The equations of this model are described below. A description of the membrane mechanisms included in the model and evidence for their expression in SPN are given in Table A1. Equations were solved using NEURON's multiorder variable time-step integration method, with a time-step of 25 μs.

**Table A1.** Ionic mechanisms included in the MVC SPN model

Transmembrane Current	Notes
Transient rectifier (*I*_A_)†	Found in majority of SPN (Whyment et al. 2011; Stalbovskiy et al. 2014), the relatively slow channel kinetics have been documented in detail (Bordey et al. 1995; Sah and McLachlan 1995; Miyazaki et al. 1996; Whyment et al. 2011); involved in AHP (Yoshimura et al. 1986b; Wilson et al. 2002).
Fast-sodium (*I*_Na3_) *	SPN action potentials are TTX sensitive (Yoshimura et al. 1986b; Wilson et al. 2002). The upstroke of action potentials initiated by a fast voltage sensitive Na^+^ current, so we employed Nav3 in the model.
Delayed rectifier (*I*_DR_)†	This sustained outward current is activated by depolarizing step commands from rest and is sensitive to TEA (Miyazaki et al. 1996; Wilson et al. 2002); active in action potential repolarization.
Ca^2+^-dependent K^+^ (*I*_AHP_)†	The AHP in SPNs has two components (fast and slow), both are calcium sensitive and one is also voltage sensitive (Yoshimura et al. 1986b; Miyazaki et al. 1996; Spanswick and Logan 1990); therefore, both were included in the model.
*V*_m_- and Ca^2+^-dependent K^+^ (*I*_K,Ca_)*	
N-type Ca^2+^ (*I*_CaN_)†	SPNs have a prominent shoulder on the downstroke of action potentials, indicating the existence of voltage-sensitive Ca^2+^ channels. In the presence of TTX, this Ca^2+^ conductance gives rise to a broad, high-threshold spike (Wilson et al. 2002) that is abolished by Co^2+^ (Yoshimura et al. 1986b) and is important for the AHP. Because the specific channel carrying this current is unknown, both persistent (L-type, *I*_CaL_) and inactivating (N-type, *I*_CaN_) Ca^2+^ currents were therefore included in the model.
L-type Ca^2+^ (*I*_CaL_)†	
Ca^2+^ handling‡	Intracellular calcium dynamics were modeled including buffering, diffusion, and Ca^2+^ pumps (cadifus.mod; Lawrence et al. 2006).

TTX, tetrodotoxin; TEA, tetraethylammonium.

* Mechanism from Migliore et al. (2001) (ModelDB Accession No. 3167).

† Mechanism from Migliore et al. (1995) (ModelDB Accession No. 3263).

‡ Mechanism from Lawrence et al. (2006) (ModelDB Accession No. 102288).

#### Governing equations.

The governing equation for membrane potential (*V*_m_) dynamics in the soma is

$$\frac{dV_{\text{M}}}{dt}=-\left(I_{\text{DR}}+I_{\text{Na3}}+I_{\text{A}}+I_{\text{Pas}}+I_{\text{AHP}}+I_{\text{K,Ca}}+I_{\text{CaN}}+I_{\text{CaL}}\right)$$

The equations describing the ionic currents are given below. All gating variables *X* have dynamics governed by

$$\frac{dX}{dt}=\frac{X-X_{\infty }}{\tau_{X}}$$

where *X*_∞_ and τ_*x*_ are functions of *V*_m_ and/or ionic concentrations.

#### Fast voltage-activated sodium current, I_Na3_.

The fast voltage-activated sodium current has the form

$$I_{\text{Na3}}={\overset{¯}{g}}_{\text{Na}3}m^{3}\text{hs}\left(V_{\text{M}}-E_{\text{Na}3}\right)$$

where *ḡ*_Na*3*_ = 50 mS/cm^2^ and *E*_Na*3*_ = 55 mV. The gating variables have functions as in Migliore et al. (2001);

$$\begin{array}{cc} s_{\infty } & =f+a_{z}\left(1-f\right)\\ \tau_{s} & =\frac{\beta \left(12,0.2,-60\right)}{3\times 10^{-4}\left[1+\alpha \left(12,-60\right)\right]}\\ \tau_{h} & =\frac{2^{0.2}}{g_{+}\left(-45,0.03,1.5\right)+g_{-}\left(45,0.03,1.5\right)}\\ h_{\infty } & =\frac{1}{1+\exp\left(\frac{V_{M}+50}{4}\right)}\\ \tau_{m} & =\frac{2^{0.2}}{g_{+}\left(-50,0.4,7.2\right)+g_{-}\left(20,0.124,7.2\right)}\\ m_{\infty } & =\frac{g_{+}\left(-50,0.4,7.2\right)}{g_{+}\left(-50,0.4,7.2\right)+g_{-}\left(-50,0.124,7.2\right)} \end{array}$$

where the function α is given below (in *I*_A_ description) and

$$\begin{array}{cc} f & =\frac{1}{1+\exp\left(\frac{V_{\text{M}}+58}{2}\right)}\\ g_{+}\left(a,b,q\right) & =\frac{a\left(V_{\text{M}}-b+\varepsilon \right)}{1-\exp\left(\frac{-V_{\text{M}}+b+\varepsilon }{q}\right)}\\ g_{-}\left(a,b,q\right) & =\frac{a\left(-V_{\text{M}}+b+\varepsilon \right)}{1-\exp\left(\frac{V_{\text{M}}+b+\varepsilon }{q}\right)} \end{array}$$

The original model of a pyramidal cell (Migliore et al. 2001) had a resting membrane potential of −65 mV. Given the resting potential of SPN = −55 mV, the parameter ε = −10 mV was introduced into the equations for the activation gating variable *m*.

#### Delayed rectifier, I_DR_.

The delayed rectifier potassium current I_DR_ has the form

$$I_{\text{DR}}={\overset{¯}{g}}_{\text{DR}}n^{3}l\left(V_{\text{M}}-E_{\text{K}}\right)$$

where the parameters are *ḡ*_DR_ = 10 mS/cm^2^ and *E*_K_ = −90 mV. The gating variable functions are as derived by Migliore et al. (1995).

#### Leak current, I_Pas_.

The leak current has Ohmic form

$$I_{\text{Pas}}={\bar{g}}_{\text{L}}\left(V_{\text{M}}-E_{\text{L}}\right)$$

where *ḡ*_L_ = 0.018 mS/cm^2^ and *E*_L_ = −40 mV.

#### AHP current, I_AHP_.

The AHP current has the form

$$I_{\text{AHP}}={\bar{g}}_{\text{AHP}}w\left(V_{\text{M}}-E_{\text{K}}\right)$$

where *ḡ*_L_ = 0.1 mS/cm^2^ and *E*_K_ = −90 mV and gating variable *w* has functions as derived in Migliore et al. (1995).

#### Ca^2+^-activated K^+^, I_K,Ca_.

The Ca- and *V*_m_-activated K^+^ current has the form

$$I_{\text{K,Ca}}={\bar{g}}_{\text{K,Ca}}o\left(V_{\text{M}}-E_{\text{K}}\right)$$

where *ḡ*_K,Ca_ = 5 mS/cm^2^ and *E*_K_ = −90 mV. The gating variable *o* has functions derived in Migliore et al. (1995).

#### L-type voltage-activated Ca^2+^ current, I_CaL_.

The L-type current has the form

$$I_{\text{CaL}}=-{\overset{¯}{g}}_{\text{CaL}}m^{2}\left(\frac{0.001}{0.001+{\left[{\text{Ca}}^{2+}\right]}_{i}}\right)12.5\left(1-\frac{{\left[{\text{Ca}}^{2+}\right]}_{i}}{{\left[{\text{Ca}}^{2+}\right]}_{o}}\right)\exp\left(\frac{V_{\text{M}}}{12.5}\right)\text{erf}\left(\frac{V_{\text{M}}}{12.5}\right)$$

where *ḡ*_CaL_ = 1 mS/cm^2^ and the functions are as in Migliore et al. (1995):

$$\tau_{m}=\frac{1}{a+b}$$

$$m_{\infty }=a\tau_{m}$$

where

$$a=\frac{15.69\left(-V_{\text{M}}+81.5\right)}{\text{exp}\left(\frac{-V_{\text{M}}+81.5}{10}\right)-1}$$

$$b=0.29\exp\left(\frac{-V_{\text{M}}}{10.86}\right)$$

#### N-type voltage-activated Ca^2+^ current, I_CaN_.

The N-type current is given in Migliore et al. (1995). The current

$$I_{\text{CaN}}=-{\overset{¯}{g}}_{\text{CaN}}m^{2}h\left(\frac{0.001}{0.001+{\left[{\text{Ca}}^{2+}\right]}_{i}}\right)12.5\left(1-\frac{{\left[{\text{Ca}}^{2+}\right]}_{i}}{{\left[{\text{Ca}}^{2+}\right]}_{o}}\right)\exp\left(\frac{V_{\text{M}}}{12.5}\right)\text{erf}\left(\frac{V_{\text{M}}}{12.5}\right)$$

$$\tau_{m}=\frac{1}{a_{m}+b_{m}}$$

$$m_{\infty }=a_{m}\tau_{m}$$

$$\tau_{h}=\frac{1}{a_{h}+b_{h}}$$

$$h_{\infty }=a_{h}\tau_{h}$$

where

$$a_{m}=\frac{0.1967\left(-V_{\text{M}}+19.88\right)}{\exp\left(-V_{\text{M}}+19.88\right)-1}$$

$$b_{m}=0.046\exp\left(\frac{-V_{\text{M}}}{20.73}\right)$$

$$a_{h}=0.00016\exp\left(\frac{-V_{\text{M}}}{48.4}\right)$$

$$b=\frac{1}{\exp\left(\frac{-V_{\text{M}}+39.0}{10}\right)+1}$$

#### A-current, I_A_.

The A-current has the form

$$I_{\text{A}}={\overset{¯}{g}}_{\text{A}}nl\left(V_{\text{M}}-E_{\text{K}}\right)$$

In the WKY SPN model, *ḡ*_A_ = 12 mS/cm^2^. The reversal potential for potassium is *E*_K_ = −90 mV. The gating variables, which are also based on the A-current mechanism in Migliore et al. (1995), with modifications to fit our experimental data, have the functions:

(1) $$n_{\infty }=\frac{1}{1+\alpha \left(\zeta_{n},V_{\frac{1}{2},n}\right)}$$

$$\tau_{n}=\frac{\beta \left(-4,0.25,-45\right)}{0.02\left[1+\alpha \left(-4,-45\right)\right]}$$

(2) $$I_{\infty }=\frac{1}{1+a\left(\zeta_{l},V_{\frac{1}{2},l}\right)}$$

$$\tau_{l}=\frac{\beta \left(2,1,-67\right)}{0.0115\left[1+\alpha \left(2,-67\right)\right]}$$

where

$$\alpha \left(\zeta ,V_{\frac{1}{2}}\right)=\exp\left[\frac{0.001\cdot \zeta \left(V_{\text{M}}-V_{\frac{1}{2}}\right)F}{R\cdot 293.16}\right]$$

$$\beta \left(\zeta ,\gamma ,V_{\frac{1}{2}}\right)=\exp\left[\frac{0.001\cdot \zeta \cdot \gamma \left(V_{\text{M}}-V_{\frac{1}{2}}\right)F}{R\cdot 293.16}\right]$$

for Faraday constant *F* and gas constant *R*. The steady-state parameters have values ζ_*n*_ = −5, ζ_*l*_ = 4, *V*_½,*n*_ = −45, and *V*_½,*n*_ = −67.

### Parameter Fitting for I_A_

The characteristics of the transient rectification in SPN have been reported previously (Dembowsky et al. 1986; Miyazaki et al. 1996; Sah and McLachlan 1995; Yoshimura et al. 1987), and the underlying current was characterized in detail by Whyment et al. (2011) and Bordey et al. (1995). These data were used to fit the *V*_m_-dependent functions *X*_∞_ and τ_*X*_ for the gating variables of the A channel. The inactivation gating variable *l* required a steady-state function *l*_∞_ and time constant τ_*l*_. Similarly, the activation gating variable *n* required a steady-state function *n*_∞_ and time constant τ_*n*_. To fit these functions to experimental data, a Borg-Graham formalism of ion channel kinetics was used (Borg-Graham 1989). The steady-state half-activation was set to *V*_½,*n*_ = −45 mV and the steady-state half-inactivation *V*_½,*l*_ = −67 mV in the model. The slope factors of activation and inactivation were set to *k*_*n*_ = −5.05 mV^−1^ and *k*_*l*_ = 6.32 mV^−1^, respectively. The maximal conductance density of the A channel was given an initial value *ḡ*_A_ = 12 mS/cm^2^.

#### Assessment of the accuracy of SPN A-current simulation.

The voltage dependency of activation of the model *I*_A_ (Fig. A1*A*_*1*_) was fitted with a sigmoid function yielding a half-activation of *V*_½,*n*_ = 34.9 mV (Fig. 5*Ac*), which was in the range of values recorded in vitro [−29.0 ± 1.9 mV (Bordey et al. 1995) and −41.7 ± 5.7 mV (Whyment et al. 2011)]. The slope factor of the activation sigmoid was *k*_*n*_ = −9.6 mV^−1^, which was also close to that seen in vitro, where *k*_*n*_ = −8.2 ± 0.9 mV^−1^ (Bordey et al. 1995). Similarly the steady-state inactivation curve of the model conductance (Fig. A1, *A*_*2*_ and *A*_*3*_) showed a good match to experimental data with a half-inactivation of *V*_½,*l*_ = −66.9 mV (against −67.2 ± 3.7 mV; Whyment et al. 2011) and a slope factor *k*_*l*_ = 6.27 mV^−1^ that was within the reported range (6.1 ± 1.0 mV^−1^; Whyment et al. 2011).

Recovery from inactivation of *I*_A_ in the model followed a monoexponential time course (Fig. A1*B*). The normalized peak conductances, plotted as a function of recovery interval (Fig. A1*B*_*2*_), are in close agreement with the experimental data (replotted from Whyment et al. 2011). The decay time course of the model conductance showed reasonable agreement with the experimental values when plotted as a function of conditioning potential (Fig. A*1C*_*2*_, within 15%; Whyment et al. 2011).

These voltage-dependent functions of activation and steady-state inactivation gave rise to a “window current” (Fig. A1*A*_*3*_), having the same amplitude (*n* = l≈0.1) and occurring over the same voltage range (−70 to −40 mV) as that seen in vitro (Whyment et al. 2011). This window region covers the range of experimentally recorded resting membrane potentials (53 ± 1.2 mV) in the WKY, and signifies a slight removal of inactivation (gating variable 0.1; 10% de-inactivation), meaning that *I*_A_ is active at rest.

#### Sensitivity of model excitability to I_A_ parameters.

Implicit in the aims of the study was an investigation of the sensitivity of the model output to the parameters of *I*_A_. The investigation focused on the parameters of *I*_A_ previously seen to alter in the sympathetic postganglionic neurons in hypertension (Robertson and Schofield 1999). These include the maximal conductance density *ḡ*_A_ and the slope factor of steady-state inactivation. We therefore systematically examined how the steady-state parameters of the A-current could influence model excitability.

The parameters of activation (*V*_½,*n*_ and ζ_*n*_) and inactivation (*V*_½,*l*_ and ζ_*l*_) are given in *Eqs. 1* and *2*, along with the values fit to the WKY data. Note that the parameter ζ is related to the slope factor *k* by

$$\zeta =k\frac{F}{\text{RT}}$$

All of the parameters were capable of altering the key features of the model excitability and output (firing frequency, repolarization inflection *V*_RI_, AHP amplitude, and AHP duration; Fig. A2). However, independently varying each parameter did not consistently reproduce the pattern of experimental findings across the strains. The only parameter that was able to alter the model excitability and output in a manner consistent with the experimental data were *ḡ*_A_ (cf. Figs. 5 and 6).
